# The platform *GrowScreen*-*Agar* enables identification of phenotypic diversity in root and shoot growth traits of agar grown plants

**DOI:** 10.1186/s13007-020-00631-3

**Published:** 2020-06-23

**Authors:** Kerstin A. Nagel, Henning Lenz, Bernd Kastenholz, Frank Gilmer, Andreas Averesch, Alexander Putz, Kathrin Heinz, Andreas Fischbach, Hanno Scharr, Fabio Fiorani, Achim Walter, Ulrich Schurr

**Affiliations:** 1 Institute of Bio- and Geosciences, IBG-2: Plant Sciences, Forschungszentrum Jülich GmbH, 52425 Jülich, Germany; 2 Present Address: BASF SE, 67117 Limburgerhof, Germany; 3 Present Address: Institute of Agricultural Sciences, ETH Zürich, Universitätstrasse 2, 8092 Zurich, Switzerland

**Keywords:** *Arabidopsis*, Robotised, Imaging, Root system architecture, Screening, Non-destructive, 1001 genomes project, Hoagland solution

## Abstract

**Background:**

Root system architecture and especially its plasticity in acclimation to variable environments play a crucial role in the ability of plants to explore and acquire efficiently soil resources and ensure plant productivity. Non-destructive measurement methods are indispensable to quantify dynamic growth traits. For closing the phenotyping gap, we have developed an automated phenotyping platform, *GrowScreen*-*Agar*, for non-destructive characterization of root and shoot traits of plants grown in transparent agar medium.

**Results:**

The phenotyping system is capable to phenotype root systems and correlate them to whole plant development of up to 280 *Arabidopsis* plants within 15 min. The potential of the platform has been demonstrated by quantifying phenotypic differences within 78 *Arabidopsis* accessions from the 1001 genomes project. The chosen concept ‘plant-to-sensor’ is based on transporting plants to the imaging position, which allows for flexible experimental size and design. As transporting causes mechanical vibrations of plants, we have validated that daily imaging, and consequently, moving plants has negligible influence on plant development. Plants are cultivated in square Petri dishes modified to allow the shoot to grow in the ambient air while the roots grow inside the Petri dish filled with agar. Because it is common practice in the scientific community to grow *Arabidopsis* plants completely enclosed in Petri dishes, we compared development of plants that had the shoot inside with that of plants that had the shoot outside the plate. Roots of plants grown completely inside the Petri dish grew 58% slower, produced a 1.8 times higher lateral root density and showed an etiolated shoot whereas plants whose shoot grew outside the plate formed a rosette. In addition, the setup with the shoot growing outside the plate offers the unique option to accurately measure both, leaf and root traits, non-destructively, and treat roots and shoots separately.

**Conclusions:**

Because the *GrowScreen*-*Agar* system can be moved from one growth chamber to another, plants can be phenotyped under a wide range of environmental conditions including future climate scenarios. In combination with a measurement throughput enabling phenotyping a large set of mutants or accessions, the platform will contribute to the identification of key genes.

## Background

### Plasticity of root system architecture

Root system architecture and especially its plasticity in acclimation to variable environments is an important agronomic trait. Plants rely on modulations of their root system architecture to respond dynamically to temporal and spatial changes in soil environments, such as heterogeneously distributed resources [23]. These responses can include growth modifications of different root classes (e.g. primary, seminal, lateral or adventitious roots), branching angles and frequencies of lateral roots or length and density of root hairs. The optimal distribution of roots in a given environment allows plants to explore and compete for resources in an efficient way or even helps to survive periods of nutrient or water deficit [24, 28].

The root phenotype, which can be observed at a certain developmental stage, is the result of a complex interaction of the genotype with multiple environmental factors [12]. Characterizing the genotypic diversity is the key to elucidate functionally the genetic and physiological basis of architectural root traits. For the model species *Arabidopsis thaliana*, for example, the genome has been sequenced (*Arabidopsis* Genome Initiative [2] and many genes have been mutated so far e.g. by the Salk Institute. In addition, numerous natural accessions have arisen in contrasting climate conditions throughout the Northern Hemisphere [52]. A quantitative description of root and shoot phenotypes of mutants and accessions is fundamental to understand how plants acclimate to a changing environment and to identify genes underlying root system architecture [40].

### Non-destructive phenotyping of root and shoot systems

Non-destructive measurement methods are indispensable to quantify periodically the phenotype of the same plant at different developmental stages. Because of the hidden nature of the below ground plant organs non-destructive root phenotyping is often technically more challenging than shoot phenotyping. One option to quantify growth and geometry of root systems non-destructively is to choose a transparent medium for plant cultivation, such as agarose gels, which is a common cultivation practice for *Arabidopsis* plants (e.g. [3, 8, 18]).

Manual measuring root traits of agar grown plants is labour intensive and therefore, time consuming. A characterization of large numbers of genotypes at multiple environments will only be feasible with high throughput phenotyping systems, and the lack of such systems hampers forward and quantitative genetic studies. To overcome this limitation, new root phenotyping methods with increased capacity and throughput are crucial. In the last years, different approaches have been published for preventing manual measurements by using camera- or scanner-based imaging with different degree of automation. Typically the simplest methods rely on the operator positioning an agar-filled container (e.g. Petri dish or cylinder) manually in front of a camera or a scanner (e.g. [15, 17, 33]). Images are taken either once or continuously, from one side for 2D imaging or from different view angles for 3D reconstruction of root systems [11, 25]. To increase capacity different platforms have been developed recently which can handle automatically more than one agar-filled plate. These systems differ widely in their capacity—ranging from 2 to 36 plates. In most systems, the plates have fixed positions [30, 47, 54]. For imaging, either multiple scanners operate in parallel [1, 43, 44] or the camera is shifted from one plate to another by using moving stages or a robotic gantry system [30, 47, 54].

To be effective at the genome scale, a measurement platform must have the capacity to phenotype thousands of plants. However, the described approaches using fixed positions of the plates and moving the camera/scanner may have limitations when considering scaling up of the experiments. If the systems are expanded significantly, the time for measuring all plates with only one optical system would very likely limit the throughput considerably. To overcome this limitation multiple imaging stations working in parallel could be an option, albeit with increased costs. Another approach is to use a fixed position for the camera to which agar plates are presented with automation solutions. This approach has been established in the so-called ‘*Microphenotron*’ system, a miniaturised platform for phenotyping young *Arabidopsis* seedlings [7, 38]. In this system the plants are grown in custom-made strips positioned in 96-well microtiter plates (2.3 cm deep) which are moved by using a robot with custom-made fingers.

The approach of moving plants was also implemented in the phenotyping platform presented in this study, which uses square Petri dishes (12 × 12 cm) filled with agar for plant cultivation. For validation of the methodology, we tested if moving the plants on a daily basis has an effect on root and shoot development. In our platform, a simultaneous imaging of roots and shoots is realised. For this purpose specially modified Petri dishes are used which allow the shoot to grow outside the plate, while the roots grow inside the agar gel. Because currently the accepted cultivation system in the scientific community is to grow the plants completely inside the Petri dish [5], the aim of this study has been to test the hypothesis that positioning of the shoot inside or outside the plate has no effect on root and shoot growth and architecture. The potential of the system, ‘*GrowScreen*-*Agar*’, was assessed by quantifying phenotypic differences of 78 *Arabidopsis* accessions selected from the 1001 genomes project [52].

## Results

### GrowScreen-Agar: automated platform for root and shoot phenotyping

**Fig. 1:**
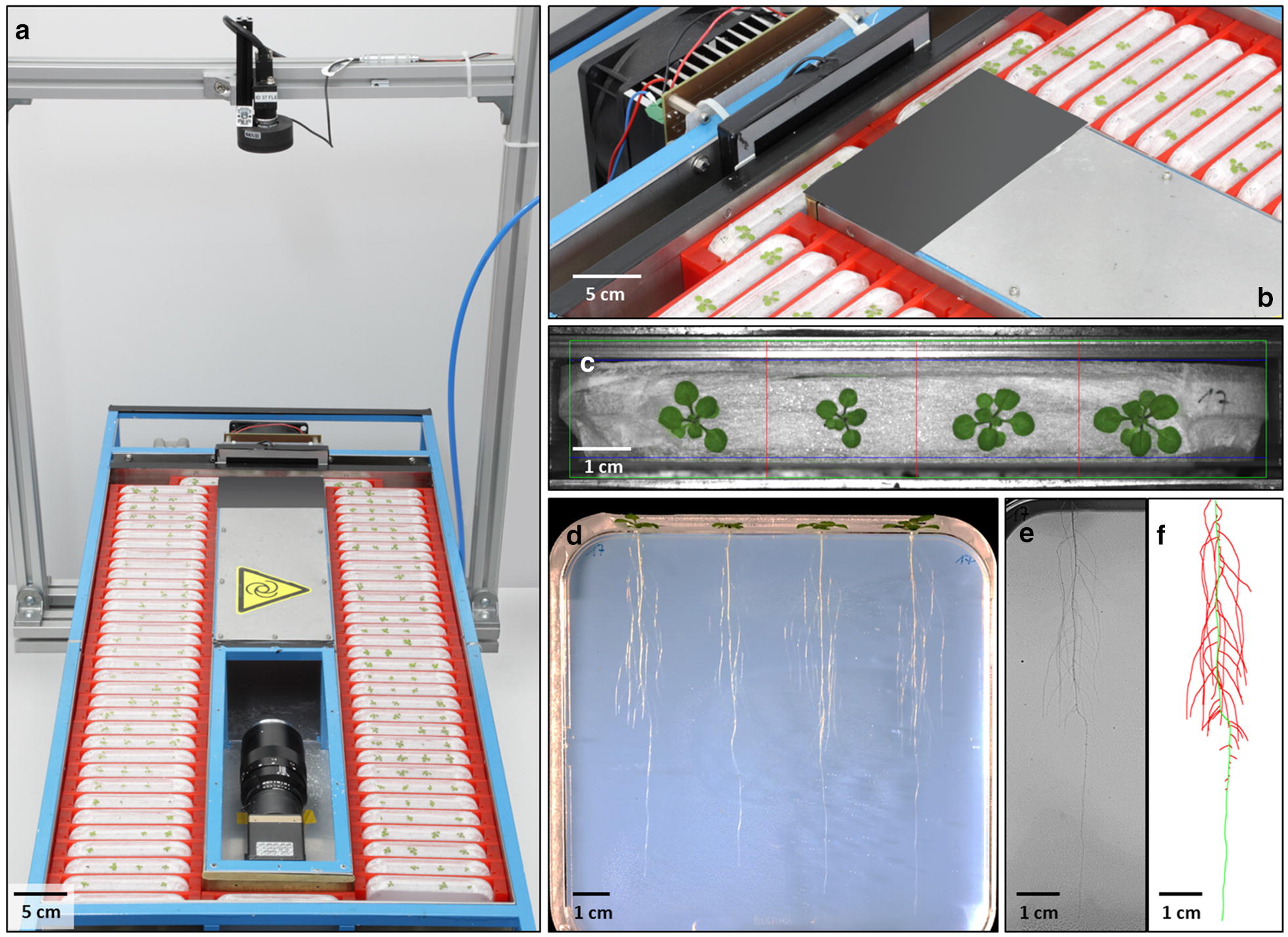
*GrowScreen*-*Agar*, mechanical setup for automated imaging roots and shoots of plants grown in agar-filled plates. The Petri dishes are fixed in red holders, which are moved in a rectangular frame by using pneumatic cylinders (**a**, **b**). At one position of the setup, Petri dishes (**b**) are optically accessible and images of root and shoot are taken (**c**, **d**). Representative original colour image of four *Arabidopsis* shoots taken by the top camera (**c**); part of an original grey scale root image taken by the bottom camera (**e**) and colour-coded image (quantified with the image-based software GROWSCREEN-ROOT) with primary root (green) and lateral roots (red) of an *Arabidopsis* plant (**f**). In total 70 Petri dishes containing up to 280 *Arabidopsis* plants fit into the *GrowScreen*-*Agar* system. During image acquisition, the opening above the bottom camera (**a**) is closed by a cover panel

The *GrowScreen*-*Agar* setup is a device for automated and non-destructive quantification of root and shoot growth and architecture of *Arabidopsis thaliana* plants or small seedlings (Fig. 1). The setup has been used in previous work [8, 57], but only minor technical details have been published before. The basis are standard square Petri dishes (12 × 12 cm) made of polystyrene (PS), which are filled with agar and placed upright to allow root growth along a vertical plane. The main difference to standard root assays (e.g. [13, 16]) is that plants are not fully enclosed within the plate with roots growing on the surface of the agar. In our system seeds are placed on the upper small side of the agar block allowing roots to grow inside the agar and shoots outside the plate through custom-made holes. This setup allows shading of the root system without shading the leaves, quantification of shoot traits including photosynthetic activity, and experimental treatment of shoots and roots in a separate way.

The holes, with a size adjusted to seed and seedling dimensions, are drilled into one side wall of the bottom part of the Petri dish (Additional file 1: Fig. S1). This side is covered with fabric tape before drilling the holes to avoid reflections of the glossy plastic surface when later taking shoot images from above. For *Arabidopsis*, up to four holes each with a diameter of 2.0 mm are evenly distributed. For filling the hot liquid agar into the Petri dishes, the holes have to be closed temporarily with another piece of fabric tape. To prevent the lid of the Petri dish from covering the holes when it is closed, part of the lid is cut out from one edge to another with a hot wire (Additional file 1: Fig. S1). To keep the plates sterile all of these mechanical modifications are carried out under a clean bench.

After preparation of the Petri dishes, the bottom parts are laid flat in the clean bench and either completely filled with agar if the shoot should grow outside the plate or filled only up to half the height if the shoot should grow inside the plate (Additional file 1: Fig. S1). When the agar has hardened, the tape closing the holes is removed and the lids are put on the bottom part to close the Petri dishes. Thereafter, the Petri dishes are sealed with the fabric tape (Fig. 1c, d). The porous tape allows gas exchange with the layer of air between lid and agar surface, avoids water aggregation on the bottom and, at the same time, prevents to a great extent contaminations with bacteria and fungi. After sowing, the agar-filled Petri dishes are fitted upright into opaque trays in groups of 15 to minimise light reaching the roots.

For phenotyping of root and shoot traits, the Petri dishes are placed into U-shaped holders (Fig. 1a, b, Additional file 1: Fig. S2) to fix them and make them transportable within the automated *GrowScreen*-*Agar* setup. The Petri dishes within the U-shaped holders are placed manually into a custom-built system (Additional file 1: Table S1 and Figs. S3, S4, overall outer dimensions: 1450 × 640 × 950 mm). The holders are moved in a rectangular conduit by using four pneumatic cylinders. Two of these cylinders (Additional file 1: Table S1) are mounted at each edge of the rectangle in direction of the stacks to push holders forward by 35 mm steps (Fig. 1a, Additional file 1: Figs. S5, S6). Another two (Additional file 1: Table S1) are sitting below the short paths of the rectangle and move two holders each sideways by 150 mm. Each plate is moved via two steps of movement (forward + sideways) which rotates the whole stack of plates counterclockwise (Additional file 1: Fig. S6). The steel sheet (Additional file 1: Fig. S4) on which the U-shaped holders are moved is slightly greased to allow an almost frictionless movement. All 70 holders have to be placed into the *GrowScreen*-*Agar* setup, even if an experiment contains less than 70 Petri dishes, to allow the pneumatic cylinders to move the whole stack of holders.

In the middle of one short side of the system, Petri dishes are optically accessible and imaged automatically. The root systems are recorded via a monochrome CCD-camera (as reported previously in [8]; Additional file 1: Table S1), which is mounted at a distance of 65 cm to the Petri dish surface, in the centre of the rectangular conduit to have a straight view through the transparent Petri dish (Fig. 1a). For illumination of the roots a white LED panel is used (Additional file 1: Table S1). The panel is mounted at a distance of 25 mm (LED panel) or 63 mm (if an infrared panel is used) from the Petri dishes to minimise visibility of water droplets condensing within the plate (Fig. 1b, d). For shoot imaging, a 2 MP camera (Additional file 1: Table S1) and a white LED ring is mounted 52 cm above the shoot (Fig. 1a, c). This optical setup has a resolution of 39 µm/px in root images (image size 10.4 MB, bmp) and of 83 µm/px in shoot images (image size: 3.56 MB, tif). The root and shoot images are labeled with a time stamp, the plate No and with ‘root’ or ‘shoot’, respectively and stored locally in a folder labeled with the imaging date. Illumination for shoot and root is switched on automatically and synchronised with each camera during image acquisition. After taking root and shoot images of one Petri dish all holders are moved one position further, to place the next Petri dish in the row in front of the cameras until all 70 plates are recorded. It takes approx. 15 min to acquire all images containing up to 280 *Arabidopsis* plants. The whole process is automated, visualised and controlled via LabView connecting to Compact FieldPoint devices (National Instruments Corporation, Austin, TX, USA). The LabView programme controls the pneumatic cylinders to move the Petri dishes as well as the image acquisition. Plants are phenotyped using the *GrowScreen*-*Agar* setup until roots reach the bottom of the Petri dishes (max. depth 120 mm) or the shoot rosettes exceed a diameter of 24 mm. For these reasons, the duration of experiments is limited to 3–4 weeks for *Arabidopsis* plants after germination.

### Image analysis of root and shoot traits

The images of whole Petri dishes are analysed using the image-based software GROWSCREEN-ROOT [33] which allows semi-automatic quantification of root system architecture (Fig. 1e, f). The key element of the software is the automatic extraction of a tree model for the root system (for more technical details see [31, 33]. Scratches on the agar gel or condensation on the inside lid of the Petri dish or water droplets on the agar (due to water loss of the agar into the air space within the plate) may negatively affect the automatic feature extraction if the scratch or water drops are located close to a root or in the same orientation as the roots. As a result, we adapted the software to allow manual corrections of artefacts that are detected as roots or of roots that could not be detected automatically. For quantitative analyses, the extracted root system can be corrected by retracing of root axes–if necessary–via a graphical user interface and by using graphics tablets with pens (Wacom Cintiq 21UX, CANCOM Deutschland GmbH, Düsseldorf, Germany). After checking or correcting the images, the software GROWSCREEN-ROOT automatically computes the root traits, which are listed in Table 1. As the phenotyping system *GrowScreen*-*Agar* enables the measurement of the same plant repeatedly at a user defined frequency (hours, days or weeks), all traits can be quantified at a single time point or in a time-course.

**Tabel 1:** Root traits of plants grown in agar-filled Petri dishes measured non-destructively with the phenotyping system *GrowScreen*-*Agar*

Root traits	Primary data
Length primary root	Length of primary root (mm)
Length lateral roots	Length of lateral roots branched from primary root (mm)
Length total roots	Sum of primary and lateral root lengths (mm)
Number lateral roots	Number of lateral roots branched from primary roots
Lateral root density	Number of lateral roots per primary root length (mm^−1^)
Branching angle	Angle between primary and branched lateral roots (°)
Diameter primary root	Average diameter of primary root (µm)
Diameter lateral roots	Average diameter of lateral roots (µm)
Root system depth	Maximum vertical depth of whole root system (mm)
Root system width	Maximum horizontal width of whole root system (mm)
Root area	Convex hull area, measured by encompassing a root system with the shortest line (mm^2^)
Root length density	Total root length per agar surface area (mm mm^−2^)
RGR primary root	Relative growth rate of primary root (% day^−1^)
RGR lateral roots	Relative growth rate of lateral roots (% day^−1^)
RGR total roots	Relative growth rate of whole root system (% day^−1^)
Ratio lateral/primary root	Ratio between length of lateral roots and primary root
Ratio primary/rooting depth	Ratio between primary root length and root system depth

To evaluate accuracy of the software tool for analysing growth and geometry of roots grown in agar, reference objects (thin metal rods) with defined lengths were inserted into the agar. A strong linear correlation (R^2^ = 0.9998) between the known length and the length of those objects quantified with the software GROWSCREEN-ROOT shows the high precision of this image-based tool and its value for extraction of digital traits.

For quantification of projected leaf area, custom-made algorithms were used that allow segmentation using thresholds of the parameters hue, saturation and colour value, and therefore, distinguishing between plant and background by creating binary masks (for more details see [34, 51].

### Validation experiments

To validate the phenotyping system, we analysed the possible effect of movement on root and shoot development using *Arabidopsis* Col-0 plants by comparing traits between plants that were moved or not moved during the experiment (experiment 1). In a second experiment, we quantified the development of roots and shoots of Col-0 plants cultivated with shoots either inside or outside the Petri dish (experiment 2). Furthermore, we assessed the potential of the phenotyping system by quantifying phenotypic differences between 78 *Arabidopsis* accessions, selected from the genotypes of the 1001 genomes project [52]; experiment 3).

### Root and shoot development is practically unaffected by moving plants regularly within the automated system

**Fig. 2:**
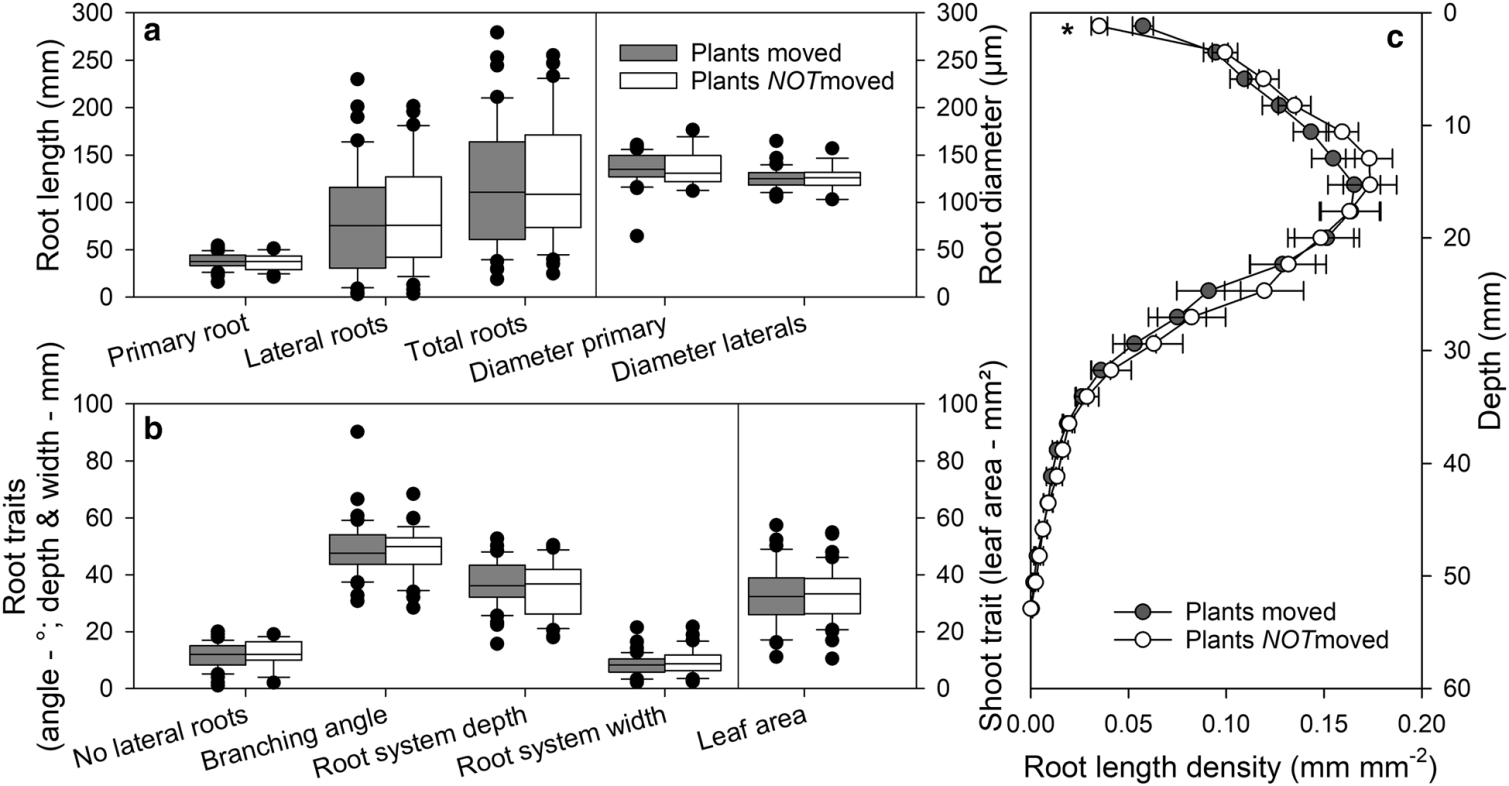
Validation of phenotyping system: Root and shoot traits of daily imaged plants (‘Plants moved’) were compared with traits of plants which were not moved for 3 weeks after germination (‘Plants *NOT*moved’). Daily imaging and therefore movement of the plants in the phenotyping system has almost no effect on growth and development of roots and shoots of *Arabidopsis* Col-0 plants. Box plots represent the distribution of values of each trait; median, 25th and 75th percentiles and extremes are shown (**a**, **b**). The line plot represents the spatial distribution of roots (**c**; mean value ± SE, n = 37–40). *indicates significant difference between the moved and not moved plants (*P *< 0.05)

For imaging roots and shoots in the presented phenotyping platform, the plants are transported automatically to the imaging position by using pneumatic cylinders. Although the friction during the movement of the plates is minimal, this procedure might result in small vibrations of the plants. In experiment 1, we tested the effect of moving the plants on root and shoot development. Plants, which were imaged daily in the *GrowScreen*-*Agar* system, were compared with plants, which were not moved over 3 weeks after germination and only imaged once at the end of the experiment (Fig. 2). No significant differences in the quantified root and shoot traits were found between moved and not moved plants (Fig. 2a, b). Moved and not moved plants exhibited similar values in the measured global root traits, such as total root length and spatial distribution of roots, such as maximal depth and width of the whole root system, as well as the distribution of root length density (Fig. 2a–c). We only found a significant difference (P < 0.05) in the top 2 mm of the agar gel in which not-moved plants produced fewer roots than moved plants (Fig. 2c). Furthermore, daily moving of plants had no significant effect on root traits derived from individual roots, such as length and diameter of primary and lateral roots, as well as number and branching angle of laterals (Fig. 2a). These results suggest that daily imaging, and consequently, moving the plants once a day has almost no influence on development of roots and shoots and the presented phenotyping approach can be used to quantify traits reliably.

### Arabidopsis roots show high variability in lateral root formation and development

The highest variability within the measured traits–independent of moving or not moving plants–was found in the development of lateral roots (Fig. 2a). While the coefficient of variation (CV) was 22.7% for primary root development, a 1.7 times higher value was measured for number of lateral roots (38.5%) and even a three times higher value for the length of lateral roots (66.8%), respectively. The higher variability in lateral roots compared to primary root was reflected in the variability of architectural traits as well–47.3% for maximal root system width versus 24.3% CV for rooting depth. While the rooting depth is affected mostly by primary root development, the width of a root system is a result of the development of lateral roots.

### *Shoot grown inside or outside the agar*-*filled plates modifies growth and architecture of Arabidopsis roots and shoots*

**Fig. 3:**
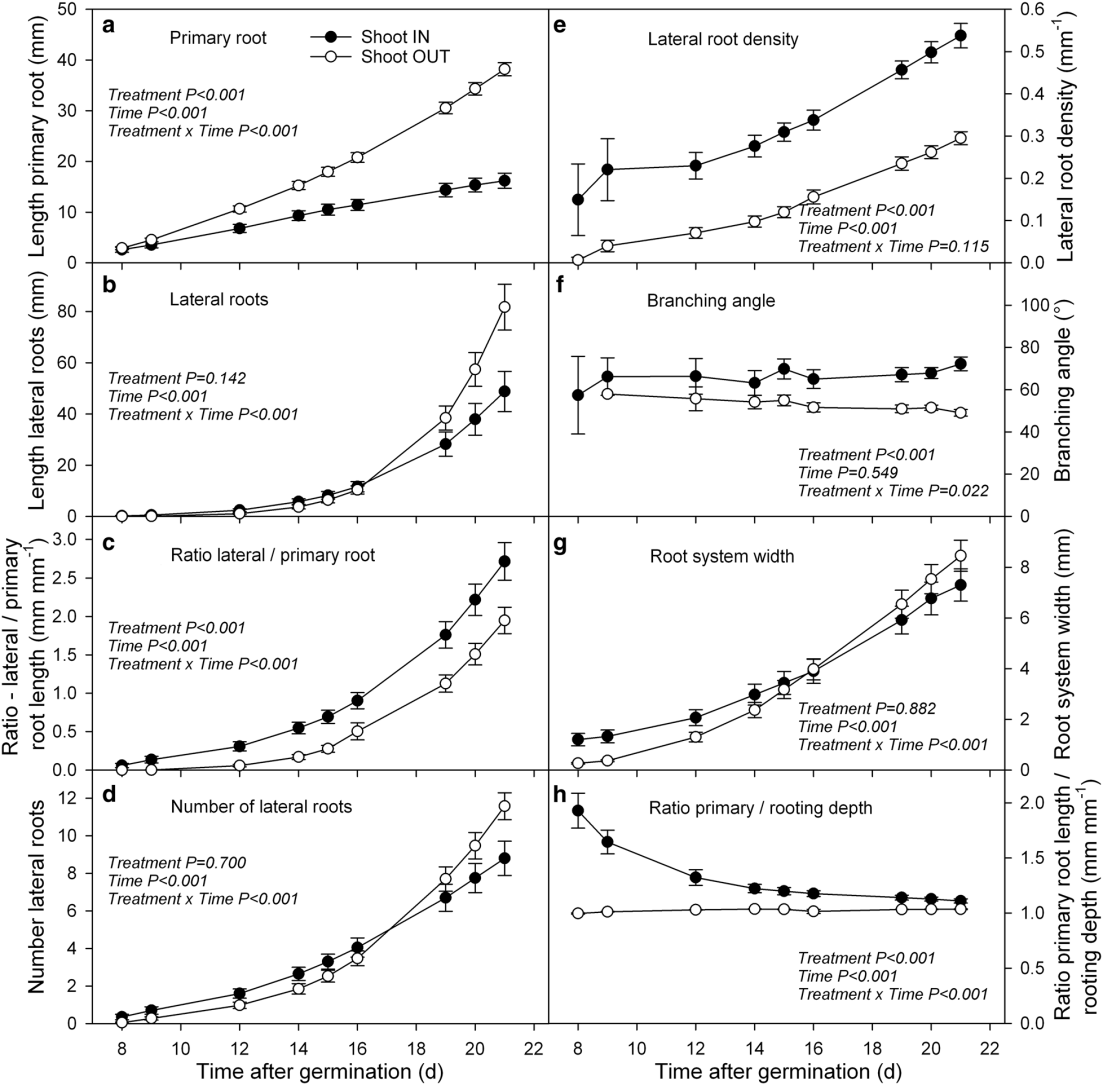
Comparison between *Arabidopsis* Col-0 plants grown with the shoot inside and outside the agar-filled Petri dish. Plants with the shoot grown inside the plate showed significant differences in the following root traits compared to plants with the leaves expanding outside the plate: length of primary (**a**) and lateral (**b**) roots, ratio between lateral and primary root length (**c**), number (**d**), root density (**e**) and branching angle (**f**) of lateral roots as well as spatial distribution of roots (**g**) and root curvature (**h**; mean value ± SE, n = 20–40)

The presented phenotyping platform *GrowScreen*-*Agar* enables simultaneous imaging of roots and shoots. The measurement of the leaf rosette is possible by using modified Petri dishes with holes, which allow the shoot to grow outside the plate, while the roots grow inside the agar medium. In experiment 2, we compared the development of *Arabidopsis* plants with the shoot inside the plate with plants with the shoot grown outside (Fig. 3). Plants were treated in the same way (same agar medium, same environmental conditions), except that the shoots grew either in the air space inside the plate or completely outside the plate. To allow the shoot to grow inside, the airspace inside a Petri dish was enlarged by filling the Petri dishes only half with agar. In contrast, in the case of the shoot outside, the Petri dishes were filled completely with agar. In both cases, the roots grew through the agar medium. To facilitate the roots to grow in the agar, the plates with the shoot inside were positioned almost horizontally for 2 days after germination. Thereafter, the plates were adjusted to the vertical position and the roots continued to grow inside the medium.

**Fig. 4:**
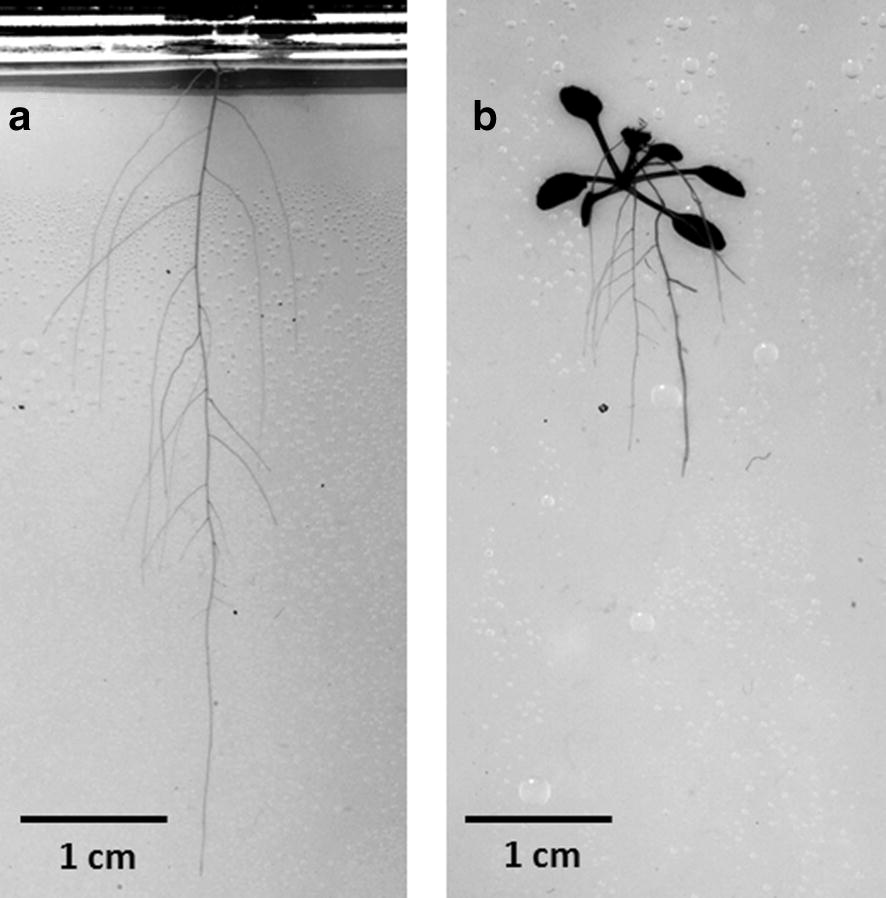
Representative original images of 3 weeks old *Arabidopsis* Col-0 plants with (**a**) shoot grown outside and (**b**) inside the agar-filled Petri dish. In contrast to (**a**) the root system under (**b**) reveals primary, lateral, and second order lateral roots, whereas under (**a**) only primary and lateral roots are developed

**Fig. 5:**
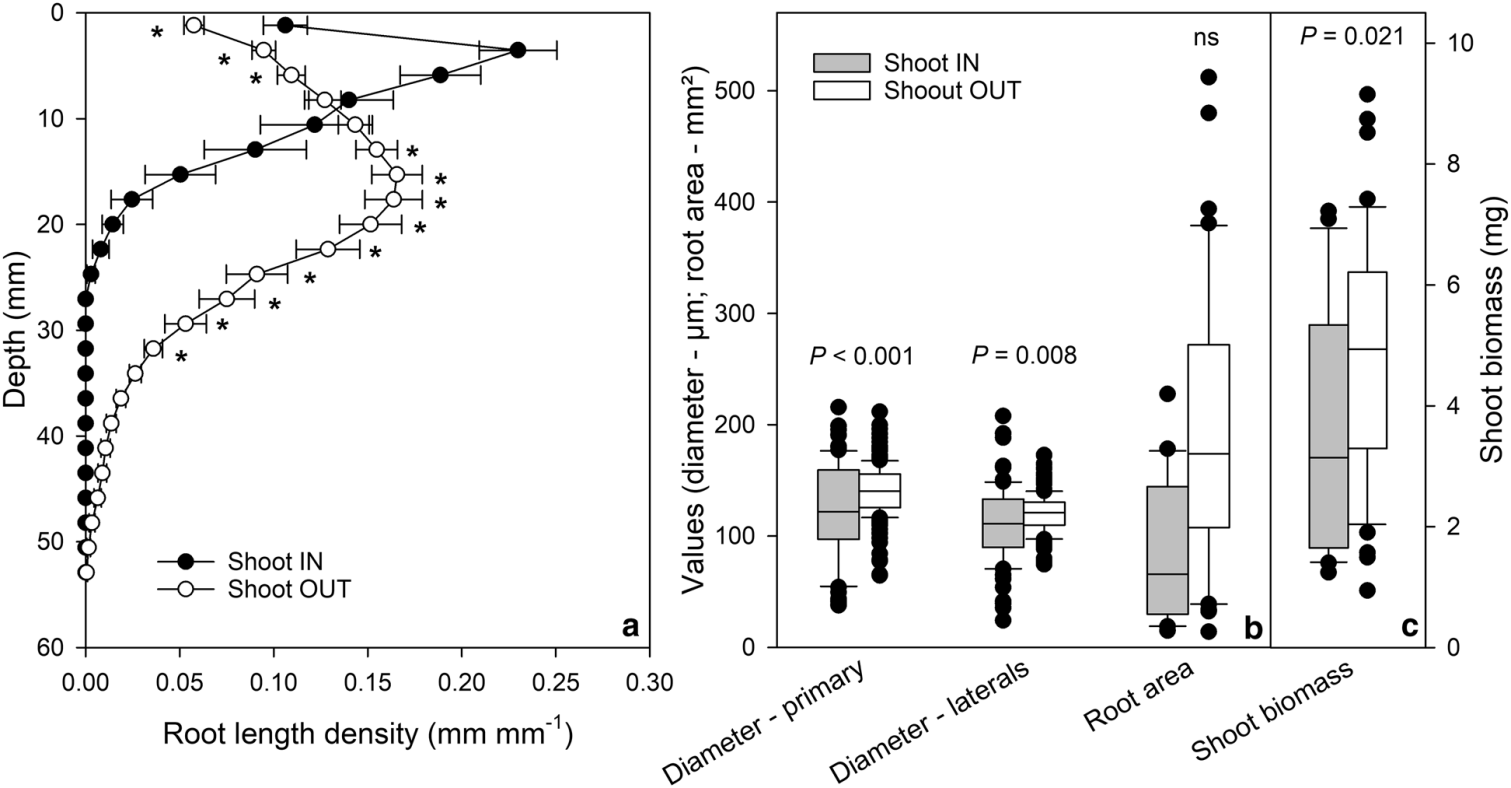
Comparison between plants grown with the shoot inside and outside the agar-filled Petri dish 3 weeks after germination. *Arabidopsis* Col-0 plants with the shoot grown inside the plate showed significant differences in the spatial distribution of roots compared to plants with the leaves expanding outside the plate (**a**; mean value ± SE, n = 20–40). Box plots represent the distribution of values of root traits (**b**) and shoot fresh weight (**c**); median, 25th and 75th percentiles and extremes are shown. * indicates significant difference between plants with the shoot inside and outside the plate (*P *< 0.05)

In general, the roots and shoots grew faster when the shoots were growing outside the plate compared to plants with shoots inside (Figs. 3, 4, 5). Significant differences between both cultivation systems could be found for shoot biomass as well as for development of primary and lateral roots (Figs. 3a, b, 5c). Three weeks after germination plants with the shoot outside the plate produced 1.3 times more shoot biomass compared to plants with the shoot inside the plate. The roots were even stronger affected, resulting in 2.4 times longer primary and 1.7 times longer lateral roots for plants with the shoot outside. While primary roots of plants with the shoot outside were already significantly longer 9 days after germination, significant differences in lateral roots in terms of length and number could not be found before day 20 (Fig. 3a, b, d). This resulted in a significantly higher ratio of lateral to primary root length (Fig. 3c; 140% at day 21) and a higher lateral root density of plants with shoot inside (Fig. 3e, 180% at day 21). Furthermore, lateral roots branched from primary roots with a significantly larger branching angle when the shoot was growing inside the plate (Fig. 3f). Compared to the other measured root traits, which changed over time, the branching angle stayed relatively stable until the end of the experiment (3 weeks after germination). Plants with the shoot inside the Petri dish exhibited a branching angle of approx. 63°, while lateral roots of plants with shoot outside branched only with an angle of 53°. However, this contrast in branching angle did not result in significant differences in the maximal width of root system between plants in both cultivation systems (Fig. 3g). The maximal horizontal distribution of a root system seems to be more reflected by growth and distribution of lateral roots than by the initial branching angle, which was measured at 0.4 mm distance from the primary root. In addition, primary roots of plants with the shoot outside the Petri dish grew almost straight downward (ratio between primary root length and rooting depth is 1; Figs. 3h, 4). In contrast, primary roots of plants growing completely inside the plate exhibited a ratio between primary root length and rooting depth of approx. 2 one week after germination indicating bending of roots. The ratio decreased over time and reached 3 weeks after germination almost 1 indicating that the primary roots grew straighter over time (Figs. 3h, 4). The initial bending of the primary root has also consequences on the distribution of root length density at different depth of the agar gel (Fig. 5a). Plants with the shoot inside the plate produced a significantly higher root length density in the upper 10 mm from the base of the root system, while below 10 mm the root length density decreased markedly. The highest root length density of plants growing completely inside the plate was found at a depth of approx. 4 mm, while the plants with the shoot outside produced the highest root length density at a depth of approx. 15 mm (Fig. 5a).

The slower root growth of plants with the shoot inside the plate goes along with significantly thinner primary and lateral roots and with a slightly, but not significantly smaller area which is covered by the roots (Fig. 5b). To summarise, plants whose shoots were grown inside the plate showed significant differences in terms of growth and architectural traits of shoots and roots compared to plants with the shoot outside the agar-filled plate. As the cultivation system with the shoot exposed to outside air enables non-destructive phenotyping of shoot traits in combination with root traits, we decided to grow the shoots outside the agar-filled plate in the following experiment.

### *Phenotyping a collection of Arabidopsis accessions shows the potential of GrowScreen*-*Agar*

**Fig. 6:**
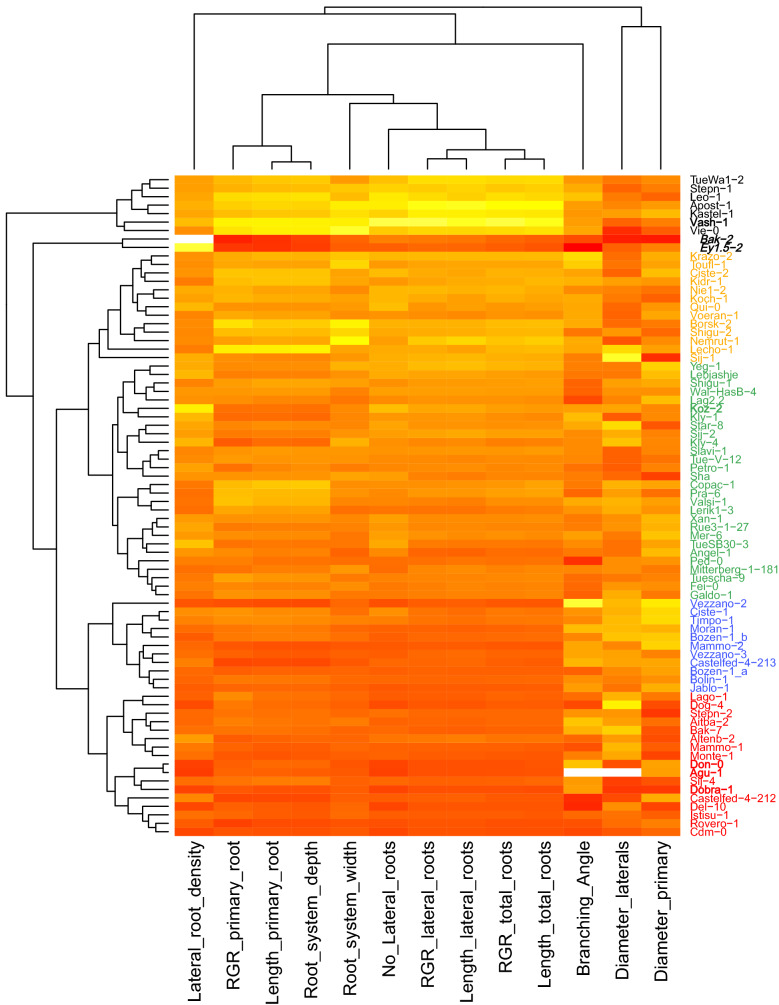
Clustering of 78 *Arabidopsis* accessions (1001 genomes project) based on variation of root traits measured at 19 days after germination. Plants were grown in agar-filled Petri dishes with the shoot outside the plate. Each column represents a trait; each row represents a genotype. Data of each sample were standardised in order to have zero mean and unit variance. The scaled value, denoted as the column Z-score, is plotted in red-yellow colour scale with red indicating low values and yellow indicating high values. White indicates data not available. Hierarchical clustering of traits and genotypes was based on the complete linkage hierarchical clustering method and Euclidean distance. Accessions belonging to the different clusters are marked in different colours (cluster 1–black, cluster 2–orange, cluster 3–green, cluster 4–blue, cluster 5–red, cluster 6–black, indent). The accessions described in the text are marked in bold letters

**Fig. 7:**
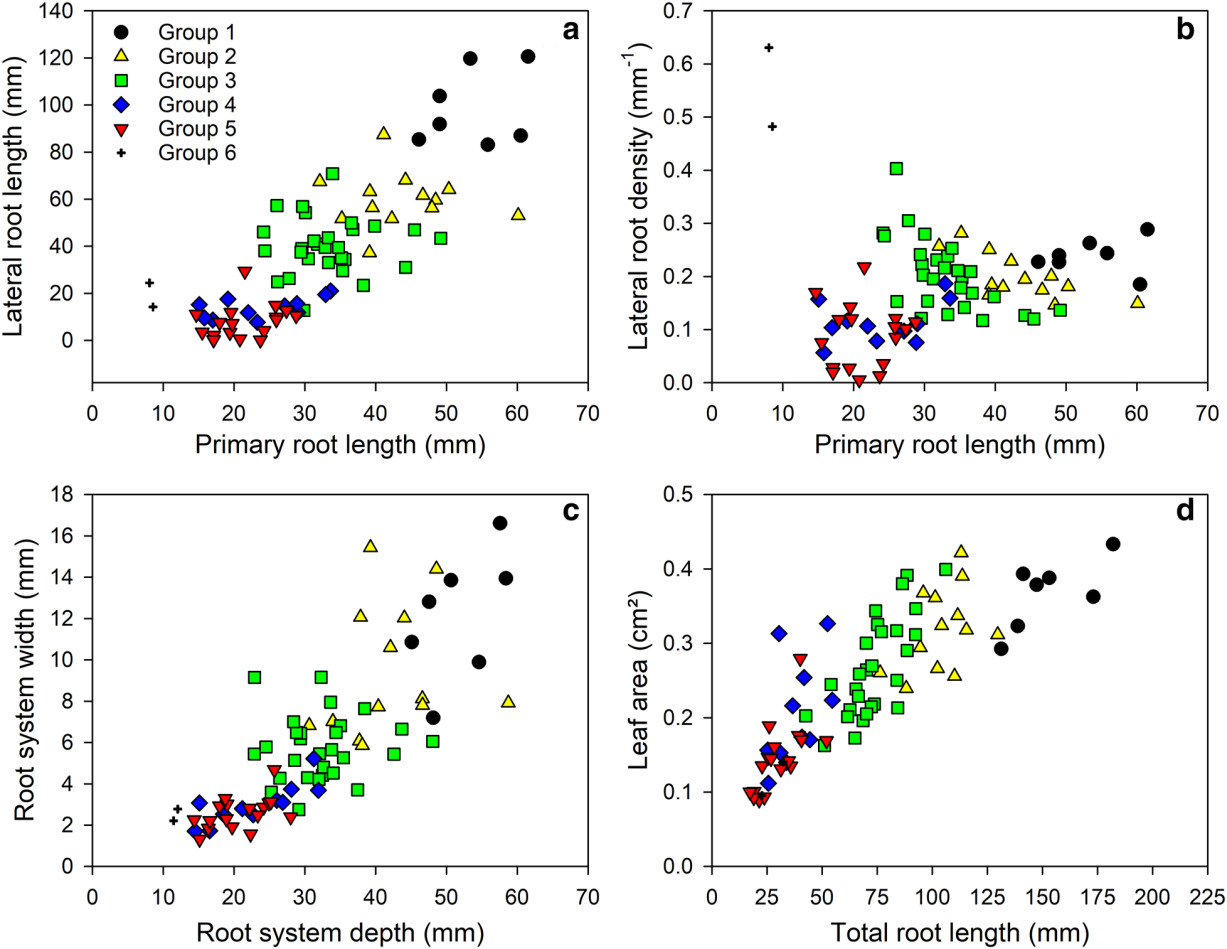
Correlation of selected root and shoot traits of 78 *Arabidopsis* accessions (1001 genomes project) measured at 19 days after germination. Plants were grown in agar-filled Petri dishes with the shoot outside the plate. Accessions belonging to the clusters (groups) identified in the hierarchical cluster analysis (Fig. 6) are marked in different colours and symbols. Mean values are shown (n = 5–13)

To demonstrate the potential and performance of our system, we have phenotyped natural variations of 78 *Arabidopsis* accessions (experiment 3) selected from the genotypes of the 1001 genomes project [52]. In total approx. 780 plants have been characterised for 3 weeks using the phenotyping platform *GrowScreen*-*Agar*. A hierarchical cluster analysis based on phenotypic traits divided the *Arabidopsis* accessions into six main clusters (Fig. 6, Additional file 1: Table S2). The six clusters that comprised between 2 and 28 genotypes were differentiated mainly by root length traits and growth of primary and lateral roots. The accessions, which clustered together to cluster No 1, had the longest primary and lateral roots while the accessions of clusters Nos 5 and 6 exhibited the shortest root systems of all 78 analysed genotypes (Figs. 6, 7). The accessions belonging to cluster Nos 2, 3 and 4 exhibited intermediate root lengths. Overall, the 78 genotypes differed in their primary root length by a factor of eight (Bak-2: 8 mm, cluster No 6 vs. Vash-1: 62 mm, cluster No 1). The variation in lateral root development was even larger. The number of lateral roots varied between the genotype with the lowest numbers of lateral roots (Agu-1, cluster No 5) and the genotype with the highest number (Vash-1, cluster No 1) by a factor of 108. In total, the variation is larger in the quantified root than in shoot traits. The accessions Vash-1 (cluster No 1) produced an 11 times larger total root length compared with the smallest accession Dobra-1 (cluster No 5). In contrast, for leaf area only a factor 5 was found between the largest (Vas-1, cluster No 1) and the smallest accession (Agu-1, cluster No 5; Fig. 7d). Of note, the two genotypes (Bak-2 and Ey1.5-2) representing cluster No 6 showed highest lateral root density although both accessions produced a relatively small root and shoot system compared the other accessions (Fig. 7a–d).

The hierarchical cluster analysis revealed strong correlations between the length of primary roots and the root system depth as well as between the length of lateral roots and the total root length (Fig. 6). The loosest correlation was found between root diameter, branching angle and lateral root density with all root length traits. In contrast, we have found positive correlations between the length of the primary roots and the length and number of lateral roots branched from primary roots (Fig. 7a, b). A similar correlation was also found between the size of a root system and the distribution of roots into vertical and horizontal orientation (Fig. 7c). To summarise, plants with longer primary roots produced more and longer lateral roots (Fig. 7a, b), encompassed a larger area with their root system (Fig. 7c) and produced a larger leaf area (Fig. 7d). However, phenotyping the 78 accessions revealed candidates, which do not match this general behaviour. For example, we have found two accessions (Don-0 and Koz-2), which exhibited a similar length of primary root and comparable root system depth, but differed widely in many other traits, especially in the number (26 times) and density (16 times) of lateral roots. We found even larger differences in the length of lateral roots. Koz-2 exhibited 57 mm long lateral roots, which is 420 times more than Don-0 with an average lateral root length of 0.14 mm. In addition, both accessions differed in shoot traits as well, resulting in a 3.4 times larger leaf area of Koz-2 compared to Don-0. These results show the potential of the presented phenotyping platform *GrowScreen*-*Agar* to quantify phenotypic differences and to identify genotypes, which differ in their shoot and root architectural traits, and therefore, could be interesting candidates for further analysis.

## Discussion

### GrowScreen-Agar enables quantitative phenotyping

The presented study aimed to develop and validate a non-destructive phenotyping system for studying dynamic traits of plants grown in transparent agar-filled Petri dishes. Our results indicate that daily imaging, and consequently, moving the plants once a day has only a negligible effect on development of roots and shoots (Fig. 2). It could be demonstrated that the presented phenotyping platform can be used to reliably quantify root and shoot phenotypic traits. The platform works non-destructively, and therefore, allows to record time series of *Arabidopsis* plants (Fig. 3). It enabled measuring phenotypic diversity within 78 *Arabidopsis* accessions of the 1001 genomes project (Figs. 6, 7). So far, the accessions of the 1001 genomes project were mainly phenotyped for shoot traits [40, 50]. In the top 10 list of the public database for *Arabidopsis* phenotypes, AraPheno (https://arapheno.1001genomes.org) shoot traits, such as days after flowering and seed weight are listed, but root traits less often presented [40]. The available root studies focus on selected accessions and traits (e.g. [39, 45], which does not allow a complete comparison of our phenotypic data with previous published data. Stetter et al. [45] for example found a large diversity in root hair traits among the investigated 166 accessions which partly overlap with the 78 accession used in our study confirming the large phenotypic variation we found in root growth traits. Interestingly, the accession Aug-1 which exhibited few and short root hairs [45] produced in our study the lowest number of lateral roots, while more and longer root hairs of the accession Vash-1 correlates with a higher number of lateral roots in our case. Both lateral roots as well as root hairs contribute to an increased root surface area which can result in an optimisation of water and nutrient uptake.

For many accessions we have found positive correlations between the size of a root system and the spatial distribution of the root system. However, we could identify accessions which exhibited a similar length of primary roots and the rooting depth, but differed significantly in their lateral branching and horizontal distribution of roots. These accessions are interesting candidates for further analysis as the differences may be beneficial under certain environmental conditions, such as spatial heterogeneous distributed resources (nutrients or water).

In general, *Arabidopsis* roots showed a higher genotypic variation in lateral development compared to primary roots (Figs. 2, 7). This higher variation in formation and development of lateral roots can be explained by the developmental origin of different root types. Primary roots are already established during embryogenesis, whereas lateral roots develop post-embryonically by branching from primary roots [35, 36]. The post-embryonic lateral root development allows dynamic acclimation of the whole root system architecture over time and adequate responses of plants to fluctuations in environmental factors, such as water and nutrient availability [18, 29]. Therefore, the genotypic variation in lateral root formation and growth plays a crucial role in the ability of plants to explore efficiently soil resources. However, the presented experiments did not include a targeted modification of water or nutrient supply and the variation in root traits, especially lateral root development was measured in particular between plants of the same genotype (Fig. 2). Therefore, the observed intra-genotypic variability could rather be caused by unintentional micro-environmental perturbations and stochasticity (randomness) in root development [4, 27].

### Cultivating shoots outside the agar-filled plates allows to combine non-destructive root and shoot phenotyping

The basis of the *GrowScreen*-*Agar* system are square Petri dishes, which are filled with agar. We modified manually the Petri dishes (making holes etc.) to allow the leaves to grow outside the plate while the roots grow through the agar (Fig. 1). As it is common practice to grow *Arabidopsis* plants completely enclosed in Petri dishes we compared the development of plants with the shoot enclosed inside the plate with plants with the shoot grown outside the plate. In most cases when plants are cultivated completely inside the Petri dish, roots grow on the surface of the agar medium. However, when the shoot is grown outside in our system, the roots have to grow through the agar as the Petri dishes are almost completely filled with agar. Root growth on and through the agar may cause differences in root development due to differences in mechanical impedance. For better comparison between plants with shoot inside and outside the Petri dishes, we developed a protocol to grow the roots in the agar medium even if the Petri dish was filled only half and the shoot was inside. The root growth through the agar medium was enabled by placing the plates for 2 days horizontal, before adjusting them upright.

To summarise the results, plants whose shoots were grown inside the plate showed significant differences in terms of growth and architectural traits of shoots and roots compared to plants with the shoots outside the agar-filled plate (Figs. 3, 4, 5). The observed reduction in root growth of *Arabidopsis* plants with shoot inside confirms the published data on *Nicotiana tabacum* [32]. Nagel et al. [32] demonstrated that the growth rate of tobacco root tips is up to four times lower if the shoot is enclosed in the Petri dish. Differences in root and shoot growth between plants with the shoot inside and outside the plate were also found by Xu et al. [53]. However, Xu et al. [53] observed that plants grown completely enclosed in the Petri dish produced larger root systems and shoots. These contrasting results can be explained by the differences in plant cultivation used in these experiments. Xu et al. [53] added sucrose into the agar medium–as it is a common practice (e.g. [16])–for plants with the shoot growing inside the agar plate, but not in the agar for plants with the shoot outside. It is well known that externally supplied sucrose in the agar medium can be taken up by the plant and stimulates root, and consequently, shoot development [10, 32, 46].

The reduction in root and shoot development of plants growing in the enclosed Petri dish observed in the presented study may be caused by different reasons. One difference between both cultivation systems is the illumination of the plants. When the shoots grow outside the plate, the roots can be shaded, while, when the shoot is grown inside the plate light reaching the root cannot be avoided. Recently, it has been shown that light triggers phototropic responses, reduces root growth, but promotes the emergence of lateral roots of *Arabidopsis* plants [41, 42]. These modifications in lateral root development resulted in a higher root length density of light grown plants similar as presented in our study for plants with the shoot inside the plate (Fig. 3). Silva-Navas et al. [42] could also demonstrate that root illumination alters the ion accumulation in the roots, resulting in a reduction of potassium and sodium while increasing the uptake of iron. Together with a light-induced stimulation of the production of pigments, hormones, such as ethylene, or reactive oxygen species (ROS), light can modify root growth and architecture of root systems [14, 48, 49, 55, 56].

In addition, both cultivation setups differed in our experiment in the absolute amount/volume of agar medium available for roots. Petri dishes with the shoot inside were filled only half to give enough space for shoot development in the airspace of the plate. In contrast, Petri dishes with the shoot outside were filled completely with agar. Halving the amount of agar, results in halving of available water and nutrients as well. However, we never observed any spatial restrictions of root development due to reduced agar volume or any changes in leaf colour, suggesting that there were no spatial limitations for roots and no limitations of nutrients during the experimental period of 3 weeks, respectively.

A typical phenomenon, which can be observed when the shoot is grown for a while inside the plate, is an etiolated shoot with long petioles (Fig. 4b). The enhanced shoot elongation may be caused by a combination of a slight light reduction (shoot inside: 90 µmol m^−2^ s^−1^, shoot outside: 100 µmol m^−2^ s^−1^), a shift in the red to far-red ratio due to absorption of the plate (shoot inside: 1.03, shoot outside: 1.19) and an alteration of ethylene concentration inside the plate [37]. Other environmental factors which differ between inside and outside the plate and have an effect on plant growth are CO_2_ concentration and relative air humidity. The CO_2_ concentration was significantly reduced when the shoot grew inside the enclosed Petri dish (inside: 310 ppm, outside: 400 ppm; LICOR 7000, Fa. LICOR Corporate, Lincoln, Nebraska, US). In contrast, the air humidity increased inside the enclosed plate (100% inside vs. 50–60% rH outside). The air humidity inside the plate combined with limited air movement may result in reduction of transpiration rate [6], and consequently, limitations in shoot and root growth of plants grown inside the plate.

In summary, a cultivation system, in which the shoot can grow outside the plate offers the possibility to cultivate the plants under conditions that do not artificially penalise shoot development and allow keeping the roots in the dark and illuminating only the leaves. Furthermore, it offers the opportunity to measure leaf traits of *Arabidopsis* rosettes, such as projected leaf area non-destructively by acquiring images using a top view camera. Having access to leaves also allows for experiments which would require a direct application of substances to the leaves (e.g. growth stimulators) or which come along with monitoring of gas exchange, chlorophyll fluorescence or other parameters that need to be probed directly on the leaves.

### Perspective and challenges of GrowScreen-Agar system

Our phenotyping platform allows the screening of up to 280 *Arabidopsis* plants in one experimental run. Due to its relatively compact size, the described prototype fits into standard walk-in climate chambers allowing to grow plants under controlled environmental conditions during the whole experimental period. The only requirements inside the climate chamber to run the system are power supply and air outlets to actuate the pneumatic system.

Instead of moving the platform from one climate chamber to another, plants can be grown at different locations and transferred manually to the *GrowScreen*-*Agar* system for imaging roots and shoots. The approach has the advantage to grow plants at the same time under different climatic conditions for quantifying genotype x environment interactions. This idea has been demonstrated by phenotyping *Arabidopsis* wild type and carotenoid mutants under different light conditions (constant low light vs. high light sunflecks treatments; [8]. In addition, plants can be exposed to different nutrient or water availabilities by adding, for example, sorbitol to the agar medium [8].

Treatments, which lead to fast temperature shifts within the agar plate are challenging because they may cause condensation of water on the lid of the Petri dishes due to evaporation from the agar medium. If the water droplets are in the same optical plane as the root system, the automatic image analysis may detect the outer surface of the droplets as roots leading to artefacts that would need to be manually corrected.

Sterile working applies for all processes during preparation and handling of the agar-filled plates to avoid fungal and bacterial contamination. The holes in the Petri dish, which allow the leaves to expand outside the plate, open a potential way for fungal spores to enter the plate. However, the risk of contamination is minimised by sealing the Petri dish–except of the holes–with tape and by using an agar medium without adding sugar. Based on our experiences, in most cases, plants can be grown up to 3–4 weeks without interference of bacterial or fungal growth in/on the agar. If contaminated plates appear during the experiments, they have to be discarded to avoid cross-contamination of non-infected plates.

The water loss of the agar results in a shrinkage of the agar gel over the experimental period. Especially in the top part, more water is lost compared to the lower part of the agar due to the holes in the top, which allow the leaves to grow outside the Petri dishes. The water loss under usual experimental conditions is not too severe and does not affect plant development during a typical experimental period. However, the asymmetrical shrinking of the agar can have consequences for seedlings geminating comparatively late after sowing. Leaves of these seedlings may expand below the lid of the Petri dish due to the shrinking of the agar in the top part. Leaf growth below the lid prevents automatic shoot imaging on the one hand, but on the other hand, limits shoot development, and consequently, root development as well. A manual, careful lifting of such leaves to enable shoot growth outside the Petri dishes may cause damages on the leaves. In most cases, these seedlings exhibiting leaves below the lid of Petri dish have to be excluded from the analysis. Nevertheless, leaf growth below the lid is a rather rare event, which can be almost prevented by keeping high humidity during seedling germination.

The *GrowScreen*-*Agar* system based on square Petri dishes with a size of 12 × 12 cm can be used to follow root development of *Arabidopsis* plants up to 3–4 weeks. In addition, we were able to demonstrate that this system is also useful for quantifying root traits of young seedlings of crop species. For example, we screened several cultivars of pea (*Pisum sativum*) for up to 10 days after germination [57]. Zhao et al. [57] demonstrated the benefit of the measured seedling root traits for the prediction towards the mature root systems by using root architecture models.

## Conclusions

The phenotyping platform *GrowScreen*-*Agar* described in this work is a unique automated prototype to phenotype root and shoot traits of *Arabidopsis* plants or young seedlings of crop species grown in agar-filled Petri dishes. We were able to demonstrate that this non-destructive platform can reliably quantify dynamic responses of root systems and correlate them to whole plant development. Non-destructive phenotyping of roots and shoots is achieved by using modified Petri dishes, which allow the shoot to grow outside the Petri dishes while the roots grow inside the agar-filled plate. The advantage of this cultivation approach is not only facilitation of shoot development in rosette-like shape but also the unique option to measure the leaves non-destructively or treat the leaves in a different way than the roots, which is almost impossible using the common practice of growing whole plants within the enclosed Petri dish.

As the *GrowScreen*-*Agar* system is compact, moveable and relatively flexible in its location, plants can be phenotyped under a wide range of environmental conditions simulating different climatic regions and/or simulating future scenarios including e.g. elevated CO_2_ conditions. Together with different adjustments of nutrient and water availabilities in the agar medium there are ample possibilities for exposing roots and shoot to multiple combinations of different environmental conditions. Especially for the identification of key genes and discovery of genetic control it is essential to phenotype the root system architecture in combination with shoot traits of different genotypes under different environmental conditions, and accordingly, the presented phenotyping platform is a useful tool.

## Methods

### Germplasm

In experiment 1, the possible effect of plant movement within the automated phenotyping system on root and shoot development was quantified. In experiment 2, the aim was to compare development of plants cultivated with shoots either inside or outside the Petri dishes. In experiment 1 and 2, *Arabidopsis thaliana* Col-0 plants have been analysed, while in experiment 3 a set of 78 different *Arabidopsis* accessions, selected from the genotypes of the 1001 genomes project [52]; list of analysed genotypes see Additional file 1: Table S2; experiment 3). The accessions have been collected from eight geographic regions spanning from European Atlantic Coast to Central Asia, and from North Africa to Southern Russia. Six large regions have been chosen, Iberian Peninsula with North Africa, Southern Italy, Eastern Europe, Caucasus, Southern Russia, and Central Asia, and have been complemented with two smaller regions, Swabia, in the Southwest of Germany, and South Tyrol, in the North of Italy, representing different climatic regions [9]. Within each region seven to twelve natural inbred strains have been selected for phenotyping natural variations.

### Plant cultivation

Petri dishes (127 × 127 × 16.5 mm; item 688161, Greiner Bio-One International GmbH, Kremsmünster, Austria) were filled with sterile agar (1% w/w, A1296, Sigma-Aldrich) containing 1/3 modified Hoagland (macronutrients [20–22]), 1/3 modified Long Ashton (micronutrients [19]), and 1/3 modified Jacobson (iron [26]) solution (Additional file 1: Tables S3–5, Protocol S1). For shoots grown outside the plate (through holes in the plate), the Petri dishes were filled completely with agar, while for shoots and roots grown inside the plate, the Petri dishes were filled only half [32], Additional file 1: Fig. S1).

Seeds were surface-sterilised using 70% (v/v) ethanol solution (3 min) and 5% (v/v) sodium hypochlorite solution (10 min, with 0.5% (w/v) active chlorine and 0.05% (v/v) Tween 20, Sigma-Aldrich). After washing three times with sterile distilled water seeds were pushed gently into the agar either through the holes of the Petri dishes or inside the plate (in the top third) depending on desired shoot growth situation (outside/inside). All plates were sealed with fabric tape (Micropore, 3 M Health Care, Neuss, Germany). The plates with holes were covered with laboratory film (Parafilm) to keep humidity high during germination and early growth development. After sowing, seeds were stratified at 4 °C for 5 days.

The plants in experiments 1 and 2 were grown in a climate chamber (VB 1100 Vario, Weiss–Gallenkamp, Loughborough, UK) at 22 °C and 60% relative humidity (RH) at day and 18 °C and 50% RH at night, 8 h/16 h light/dark cycle, and a light intensity of approx. 100 µmol m^−2^ s^−1^ photosynthetically active radiation at shoot level if the shoot was grown outside the plate. If the shoot was grown completely inside the agar-filled Petri dish (experiment 2) the light intensities at shoot level was slightly lower (approx. 90 µmol m^−2^ s^−1^). In experiment 3, the 78 *Arabidopsis* accessions were exposed to similar light conditions in the climate chamber as in experiments 1 and 2, but constant day and night temperature of 15 °C, in combination with 60% RH at day and night and 12 h/12 h light/dark cycle. The plants were placed—only for imaging—inside the automated imaging platform and afterwards back into the climate chamber.

### Statistical analysis

The effect of moved/not moved plants within the automated phenotyping system (Fig. 2) and the comparison between shoots grown inside and outside the agar-filled plates (Fig. 5) was analysed using a one-way ANOVA (SigmaPlot Version 13, Systat Software Inc., Inc., San Jose, CA, USA). Two-way ANOVA for repeated measures over time was used to analyse the time by treatment interactions (Fig. 3; SigmaPlot Version 13, Systat Software Inc., Inc., San Jose, CA, USA). Post hoc comparisons of treatment effects were performed within each group using the Tukey adjustment. To quantify the variation of measured traits the coefficient of variation (CV) was calculated. Hierarchical cluster analysis was performed to visualise the data globally. The cluster analysis was conducted based on the complete linkage hierarchical clustering method and Euclidean distances, with the results visualised as a heatmap using the R “heatmap.2” function of the corresponding R package (R version 3.6.1; package vegan Fig. 6).

## Supplementary information

**Additional file 1.***GrowScreen-Agar* mechanical setup parts list and technical drawings, list of phenotyped germplasm, and description of modified Hoagland solution.

## Data Availability

The datasets generated and analysed during the current study are available in the e!DAL research data publication system, 10.25622/FZJ/2020/0. The software that was developed for these studies is available for academic partners for non-commercial purposes upon request sent to the corresponding author, provided that bilateral terms-of-use agreements can be concluded.
