# Supplementary material for: The platform GrowScreen-Agar enables identification of phenotypic diversity in root and shoot growth traits of agar grown plants
Source: Plant Methods. 2020 Jun 23;16:89. doi: 10.1186/s13007-020-00631-3 (PMC7310412; doi:10.1186/s13007-020-00631-3)
Supplement: Supplementary file 1 — Additional file 1.GrowScreen-Agar mechanical setup parts list and technical drawings, list of phenotyped germplasm, and description of modified Hoagland solution. [file 13007_2020_631_MOESM1_ESM.docx]

**Additional file 1 - *GrowScreen-Agar* mechanical setup parts list and technical drawings, list of phenotyped germplasm, and description of modified Hoagland solution.**

**Additional file 1**

**Supplement Tables**

Table S1: Parts list of the *GrowScreen-Agar* setup (see Additional File 1: Fig. S3).

| **Function** | **Component** | **Producer** | **Count** |
| --- | --- | --- | --- |
| Root imaging - camera | IPX-16M3-G, 16 MP | Imperx Incorporated, Boca Raton, FL, USA | 1 |
| Root imaging - optics | Makro-Planar T* 2/100 ZF-I lens | Carl Zeiss AG, Oberkochen, Germany | 1 |
| Root imaging - illumination | Heitronic 27295 H, 6500K white light, 130x130 mm | Vollmer GmbH, Königsbach-Stein, Germany | 1 |
| Shoot imaging - camera | 2 MP camera (GRAS-20S4C-C, 2 MP | Point Grey Research Inc., Richmond, Canada | 1 |
| Shoot imaging - optics | Cosmicar / Pentax TV 25 mm 1:1.4 lens | Ricoh Imaging Company Ltd., Tokyo, Japan | 1 |
| Shoot imaging - illumination | LED ring, LDR2-70SW2 | CCS Inc., Kyoto, Japan | 1 |
| Transport of Petri dishes | Pneumatic cylinder type 61M2P040A0050 with 40 mm bore and 50 mm stroke, double acting | Camozzi, Brescia, Italy | 2 for counterclockwise and 2 for clockwise |
|  | Pneumatic cylinder type 25N2A16A150 with 16 mm bore and 150 mm stroke, double acting | Camozzi, Brescia, Italy | 2 for counterclockwise and 2 for clockwise |
|  | Valve cluster 3P8-GAA-8M-G77 with 8x 5/2 pneumatic valve | Camozzi, Brescia, Italy | 1 |
| Mechanical setup framework | Steel frame, dimensions see Additional File 1: Fig. S4 | any | 1 |
| Frame – shoot camera | Aluminium profile 40x40x950 mm | any | 2 |
|  | Aluminium profile 40x40x640 mm | any | 3 |
| Control unit and accessories | Network Interface FieldPoint cFP-1808 | National Instruments, Texas, USA | 1 |
|  | Relay module cFP-RLY-425 | National Instruments, Texas, USA | 1 |
|  | 24V power supply | Phoenix Contact, Blomberg, Germany | 1 |
|  | Relay GMS-OAC 84130105 | Crouzet, Hilden, Germany | 4 |

Table S2: List of 78 *Arabidopsis* accessions from different geographic regions (1001 genomes project, Weigel and Mott, 2009). Six groups of accessions were identified from hierarchical clustering based on variation of root traits (Fig. 6).

| **Strain name** | **Geographic region** | **Group** (based on clustering, Fig. 6) |
| --- | --- | --- |
| Agu-1 | Iberian Peninsula/N.Africa | 5 |
| Aitba-2 | Iberian Peninsula/N.Africa | 5 |
| Altenb-2 | South Tyrol | 5 |
| Angel-1 | Southern Italy | 3 |
| Apost-1 | Southern Italy | 1 |
| Bak-2 | Caucasus | 6 |
| Bak-7 | Caucasus | 5 |
| Bolin-1 | Eastern Europe | 4 |
| Borsk-2 | Southern Russia | 2 |
| Bozen-1_a | South Tyrol | 4 |
| Bozen-1_b | South Tyrol | 4 |
| Castelfed-4-212 | South Tyrol | 5 |
| Castelfed-4-213 | South Tyrol | 4 |
| Cdm-0 | Iberian Peninsula/N.Africa | 5 |
| Ciste-1 | Southern Italy | 4 |
| Ciste-2 | Southern Italy | 2 |
| Copac-1 | Eastern Europe | 3 |
| Del-10 | Eastern Europe | 5 |
| Dobra-1 | Eastern Europe | 5 |
| Dog-4 | Caucasus | 5 |
| Don-0 | Iberian Peninsula/N.Africa | 5 |
| Ey1.5-2 | Swabia | 6 |
| Fei-0 | Iberian Peninsula/N.Africa | 3 |
| Galdo-1 | Southern Italy | 3 |
| Istisu-1 | Caucasus | 5 |
| Jablo-1 | Eastern Europe | 4 |
| Kastel-1 | Eastern Europe | 1 |
| Kidr-1 | Southern Russia | 2 |
| Kly-1 | Central Asia | 3 |
| Kly-4 | Central Asia | 3 |
| Koch-1 | Eastern Europe | 2 |
| Koz-2 | Central Asia | 3 |
| Krazo-2 | Southern Russia | 2 |
| Lag2.2 | Caucasus | 3 |
| Lago-1 | Southern Italy | 5 |
| Lebjashje | Central Asia | 3 |
| Lecho-1 | Eastern Europe | 2 |
| Leo-1 | Iberian Peninsula/N.Africa | 1 |
| Lerik1-3 | Caucasus | 3 |
| Mammo-1 | Southern Italy | 5 |
| Mammo-2 | Southern Italy | 4 |
| Mer-6 | Iberian Peninsula/N.Africa | 3 |
| Mitterberg-1-181 | South Tyrol | 3 |
| Monte-1 | Southern Italy | 5 |
| Moran-1 | Southern Italy | 4 |
| Nemrut-1 | Caucasus | 2 |
| Nie1-2 | Swabia | 2 |
| Ped-0 | Iberian Peninsula/N.Africa | 3 |
| Petro-1 | Eastern Europe | 3 |
| Pra-6 | Iberian Peninsula/N.Africa | 3 |
| Qui-0 | Iberian Peninsula/N.Africa | 2 |
| Rovero-1 | South Tyrol | 5 |
| Rue3-1-27 | Swabia | 3 |
| Sha | Central Asia | 3 |
| Shigu-1 | Southern Russia | 3 |
| Shigu-2 | Southern Russia | 2 |
| Sij-1 | Central Asia | 2 |
| Sij-2 | Central Asia | 3 |
| Sij-4 | Central Asia | 5 |
| Slavi-1 | Eastern Europe | 3 |
| Star-8 | Swabia | 3 |
| Stepn-1 | Southern Russia | 1 |
| Stepn-2 | Southern Russia | 5 |
| Timpo-1 | Southern Italy | 4 |
| Toufl-1 | Iberian Peninsula/N.Africa | 2 |
| TueSB30-3 | Swabia | 3 |
| Tuescha-9 | Swabia | 3 |
| Tue-V-12 | Swabia | 3 |
| TueWa1-2 | Swabia | 1 |
| Valsi-1 | Southern Italy | 3 |
| Vash-1 | Caucasus | 1 |
| Vezzano-2 | South Tyrol | 4 |
| Vezzano-3 | South Tyrol | 4 |
| Vie-0 | Iberian Peninsula/N.Africa | 1 |
| Voeran-1 | South Tyrol | 2 |
| Wal-HasB-4 | Swabia | 3 |
| Xan-1 | Caucasus | 3 |
| Yeg-1 | Caucasus | 3 |

Table S3: Element and molecular ion concentrations in 1% agar suspension (w/w) and 1/3 or 1/1 strength nutrient solution (n/V) used in *GrowScreen-Agar* experiments. Values are in µmol/kg agar nutrient suspension (pH 5-6) or in µmol/l nutrient solution. The concentrations of Ca, Mg and Na in chemically digested agar powder were measured using ICP-OES (Inductively Coupled Plasma with Optical Emission Spectroscopy), and of K, B, Mn, Cu, Zn, Mo and Fe using ICP-MS (Inductively Coupled Plasma Mass Spectrometry) (both: n=2, x̅ ±10-20% rel.). The concentrations of Cl^-^, NO_3_^-^, PO_4_^3-^ and SO_4_^2-^ in aqueous eluates of the agar powder were determined using ion chromatography (Metrohm IC 850 Professional) (n=2, x̅ ±5% rel.). The nutrient composition and concentration of agar are based on its natural origin as marine red algae. The element and molecular ion concentrations of 1/3 or 1/1 strength nutrient solution are calculated data.

|  | Ca | Mg | Na | K | B | Mn | Cu | Zn | Mo | Fe | Cl^-^ | NO_3_^-^ | PO_4_^3-^ | SO_4_^2-^ |
| --- | --- | --- | --- | --- | --- | --- | --- | --- | --- | --- | --- | --- | --- | --- |
| 1%  Agar | 238 | 148 | 2653 | 32.1 | 60.6 | 0.045 | 0.212 | 1.87 | <0.06 | 7.11 | 494 | 2.42 | 116 | 7.81 |
| 1/3 Nutrients | 1667 | 667 | 0.33 | 2093 | 16.7 | 3.33 | 0.33 | 0.33 | 0.17 | 30.0 | 6.67 | 5000 | 333 | 698 |
| 1/1 Nutrients | 5000 | 2000 | 1.0 | 6280 | 50 | 10 | 1.0 | 1.0 | 0.5 | 90.0 | 20.0 | 15 10^3^ | 1000 | 2094 |

Table S4: Comparison of the macro- and micronutrient concentrations in ppm in Hoagland *et al.* (1933, 1938, 1950), Jacobson (1951), and Long Ashton (Hewitt, 1966) full strength solutions with the modified solution used in *GrowScreen-Agar* experiments (Additional File 1: Table S3). The original element concentrations, which are modified for preparing the *GrowScreen-Agar* solution, are highlighted in grey.

|  | Ca | Mg | Na | K | B | Mn | Cu | Zn | Mo | Fe | Cl | N | P | S |
| --- | --- | --- | --- | --- | --- | --- | --- | --- | --- | --- | --- | --- | --- | --- |
| Hoagland 1933 | 200 | 48.6 | - | 235 | 0.11 | 0.11 | 0.014 | 0.022 | 0.018 | 1.00 | 0.14 | 210 | 31 | 64 |
| Hoagland1938 | 200 | 48.6 | - | 235 | 0.50 | 0.50 | 0.02 | 0.05 | 0.048 | 1.00 | 0.65 | 210 | 31 | 64 |
| Hoagland1950 | 200 | 48.6 | - | 235 | 0.50 | 0.50 | 0.02 | 0.05 | 0.011 | 1.00 | 0.65 | 210 | 31 | 64 |
| Jacobson 1951 | - | - | - | 10.48 | - | - | - | - | - | 5.00 | - | - | - | 2.87 |
| Hewitt 1966 | - | - | 0.023 | - | 0.54 | 0.55 | 0.064 | 0.065 | 0.048 | - | - | - | - | 0.39 |
| *Grow-Screen-Agar* | 200 | 48.6 | 0.023 | 246 | 0.54 | 0.55 | 0.064 | 0.065 | 0.048 | 5.03 | 0.71 | 210 | 31 | 67 |

Table S5: The concentrations of compounds in Milli-Q Synthesis water as solvent for the preparation of nutrient stock solutions which are used in *GrowScreen-Agar* experiments (Additional File 1: Table S4) modified according to Hoagland *et al.* (1933, 1938, 1950, stock solutions No 1-4), Jacobson (1951, stock solution No 6) and Hewitt (1966, stock solution No 5). To avoid cross-reactions and resulting precipitates the nutrients are separated into six bottles and the amounts of each element in the six stock solutions restricted. 5 ml each of stock solutions 1 and 2, 2 ml of stock solution 3, and 1 ml each of the stock solutions No 4, 5 and 6 filled up to one liter of nutrient solution provide a full strength nutrient solution*. According to Jacobson’s protocol (Jacobson, 1951) iron is available as Fe-EDTA complex in solution No 6. Solutions 7 to 10 contain the reactants for producing this chelate complex (Additional File 1: Prot. S1).

|  | Stock solution number | Mass concentration (g/l) | Molar concentration 10^-3^ (mol/l) | Stock solution / litre 10^-3^ (l) | ^*^Full nutrient conc. 10^-6^ (mol/l) |
| --- | --- | --- | --- | --- | --- |
| KNO_3_ | 1 | 101.103 | 1000 | 5 | 5000 |
| Ca(NO_3_)_2_ ∙4 H_2_O | 2 | 236.149 | 1000 | 5 | 5000 |
| MgSO_4_ ∙ 7 H_2_O | 3 | 246.475 | 1000 | 2 | 2000 |
| KH_2_PO_4_ | 4 | 136.086 | 1000 | 1 | 1000 |
| MnCl_2_ ∙ 4 H_2_O | 5 | 1.979 | 10 | 1 | 10 |
| CuSO_4_ ∙ 5 H_2_O | 5 | 0.250 | 1 | 1 | 1 |
| ZnSO_4_ ∙ 7 H_2_O | 5 | 0.288 | 1 | 1 | 1 |
| H_3_BO_3_ | 5 | 3.092 | 50 | 1 | 50 |
| Na_2_MoO_4_ ∙2 H_2_O | 5 | 0.121 | 0.5 | 1 | 0.5 |
| C_10_H_12_FeN_2_O_8_^-^ (Fe-EDTA) | 6 | 30.965 | 90 | 1 | 90 |
| FeSO_4_ ∙ 7 H_2_O | 7 | 25.021 | 90 | 1 | 90 |
| C_10_H_16_N_2_O_8_ (EDTA) | 8 | 26.302 | 90 | 1 | 90 |
| H_2_SO_4_ | 9 | 0.1962 | 2 | 1 | 2 |
| KOH | 10 | 15.710 | 280 | 1 | 280 |

**Supplement Protocol**

Prot. S1: Protocol for preparing the Fe-EDTA solution modified according to Jacobson (1951).

For synthesis of the [Fe(C_10_H_12_N_2_O_8_)(H_2_O)]^-^ complex two solutions are prepared first.

Solution (1): 25.02 g FeSO_4_ ∙ 7 H_2_O are dissolved in 300 ml Milli-Q-H_2_O and 4 ml 0.5 mol/l H_2_SO_4_ are added. For dissolving, the 1 l beaker is placed on a magnetic stirrer for 1 h at 400 rpm.

Solution (2): 26.30 g Titriplex II (EDTA) are dissolved in 300 ml Milli-Q-H_2_O and 280 ml 1 mol/l KOH added and placed on a magnetic stirrer for 1 h at 400 rpm.

For better dissolving, the above mixtures can also be slightly heated. Solutions (1) and (2) are pooled and filled up to about 950 ml with H_2_O and then transferred into a 2 l Erlenmeyer flask as solution (3). Solution (3) is vigorously aerated for 16 h by placing the Erlenmeyer flask on a magnetic stirrer and stirring at 300 rpm. Compressed air is passed through the solution (4). The Erlenmeyer flask is thereby wrapped with aluminum foil. The solution (4) is finally filled up to 1 l in a 1000 ml volumetric flask, aliquoted in brown bottles of 250 ml each and stored in a refrigerator.

The reaction schemes for producing the Fe-EDTA complex are presented hereafter. Y^4-^ stands for the acid residue (C_10_H_12_N_2_O_8_)^4-^ of the EDTA molecule (H_4_Y = C_10_H_16_N_2_O_8_).

Reactants:

FeSO_4_ ∙ 7 H_2_O → [Fe(H_2_O)_6_]^2+^ + SO_4_^2-^ + H_2_O (1)

[Fe(H_2_O)_6_]^2+^ → [Fe(H_2_O)_5_OH]^+^ + H^+^ (1)

[Fe(H_2_O)_5_OH]^+^ → Fe(H_2_O)_4_(OH)_2_ + H^+^ (1)

H_2_SO_4_ → H^+^ + HSO_4_^-^ (1)

HSO_4_^-^ → H^+^ + SO_4_^2-^ (1)

H_4_Y + 4 KOH → 4 K^+^ + Y^4-^ + 4 H_2_O (2)

Fe(H_2_O)_4_(OH)_2_ + 2 SO_4_^2-^ + 4 H^+^ + 4 K^+^ + Y^4-^ + 5 H_2_O (3)

Redox and complexation reactions:

Fe(H_2_O)_4_(OH)_2_ + 0.25 O_2_ + 2.5 H_2_O → [Fe(H_2_O)_6_]^3+^ + 3 OH^-^ (4)

[Fe(H_2_O)_6_]^3+^ + Y^4-^ → [FeY(H_2_O)]^-^ + 5 H_2_O (4)

Products:

K[Fe(C_10_H_12_N_2_O_8_)(H_2_O)] ∙ H_2_O / K_2_SO_4_ / KOH / H_2_SO_4_ / H_2_O

**Supplement Figures**

**
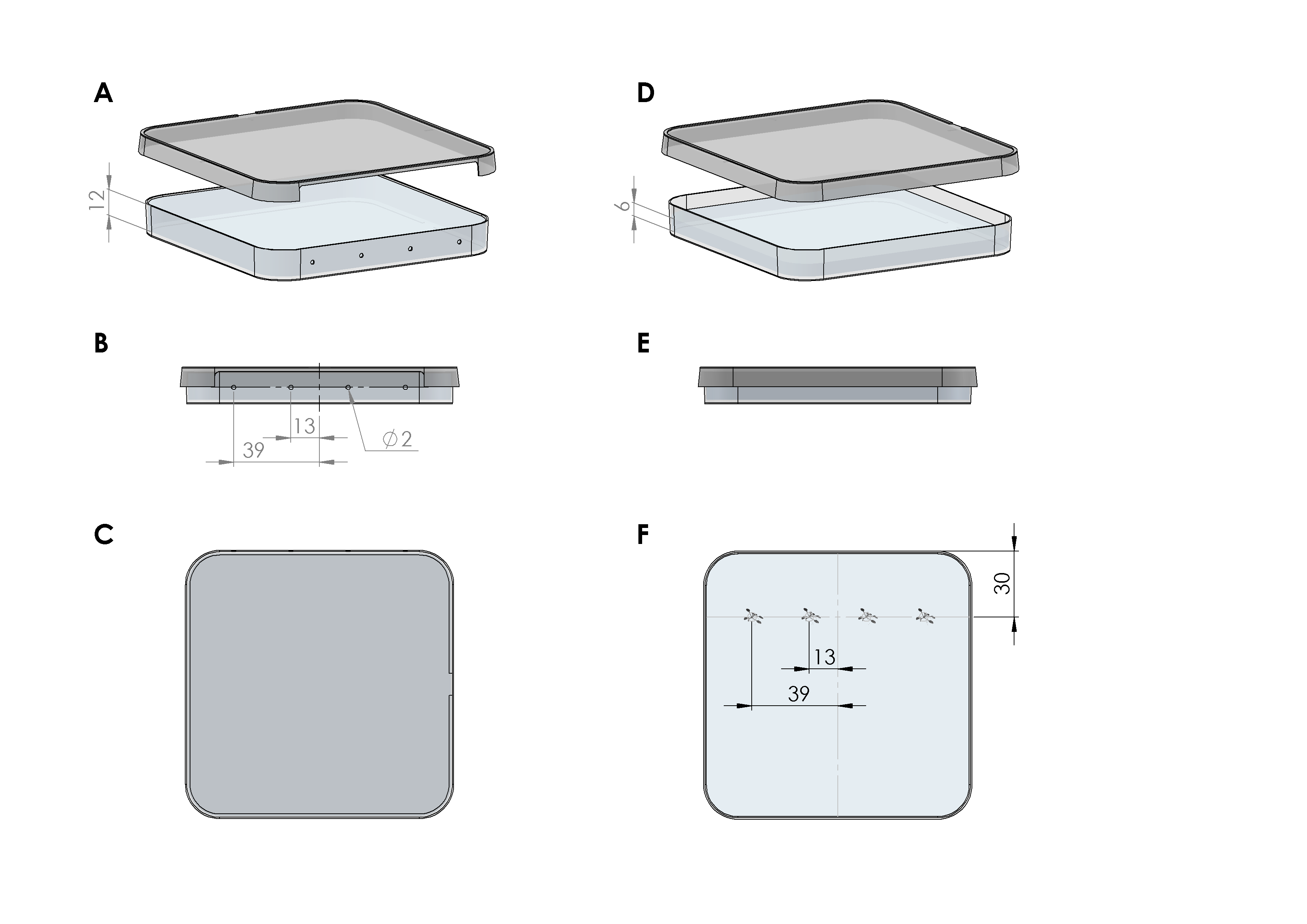
**

Fig. S1: Modified Petri dishes (A-C) have been used for phenotyping roots of *Arabidopsis* plants with the shoot grown outside the plate. A and B illustrates the position of the holes (diameter 2.0 mm) through which the shoot is developing and which part of the lid of the Petri dish has been cut out. After preparation the modified Petri dishes are filled completely with agar (A-C, thickness of the agar 12 mm).

In the case of shoots growing inside the plate (experiment 2), the Petri dishes are NOT modified and filled only half with agar (D-F, thickness of the agar 6 mm) to allow the shoot to develop in the air space inside the plate. The position at which the seeds are placed inside the plate is indicated in F.


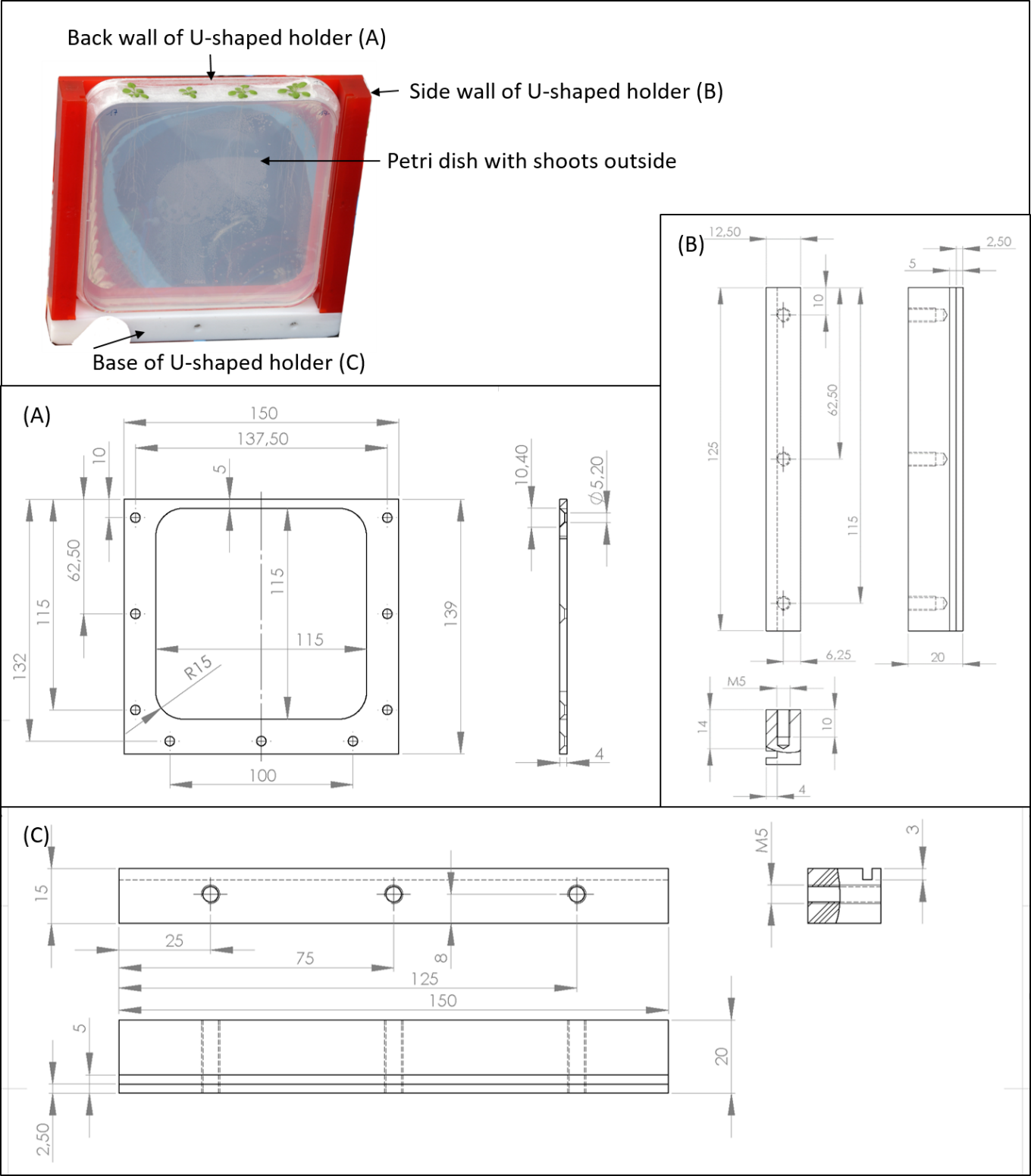


Fig. S2: The Petri dishes are placed into U-shaped holders to fix them and make them transportable within the automated *GrowScreen-Agar* setup. Original image of a U-shaped holder with a Petri dish (top) and technical drawings of the red back wall (A), the left and right red side walls (B) and the white base (C) of the U-shaped holder.


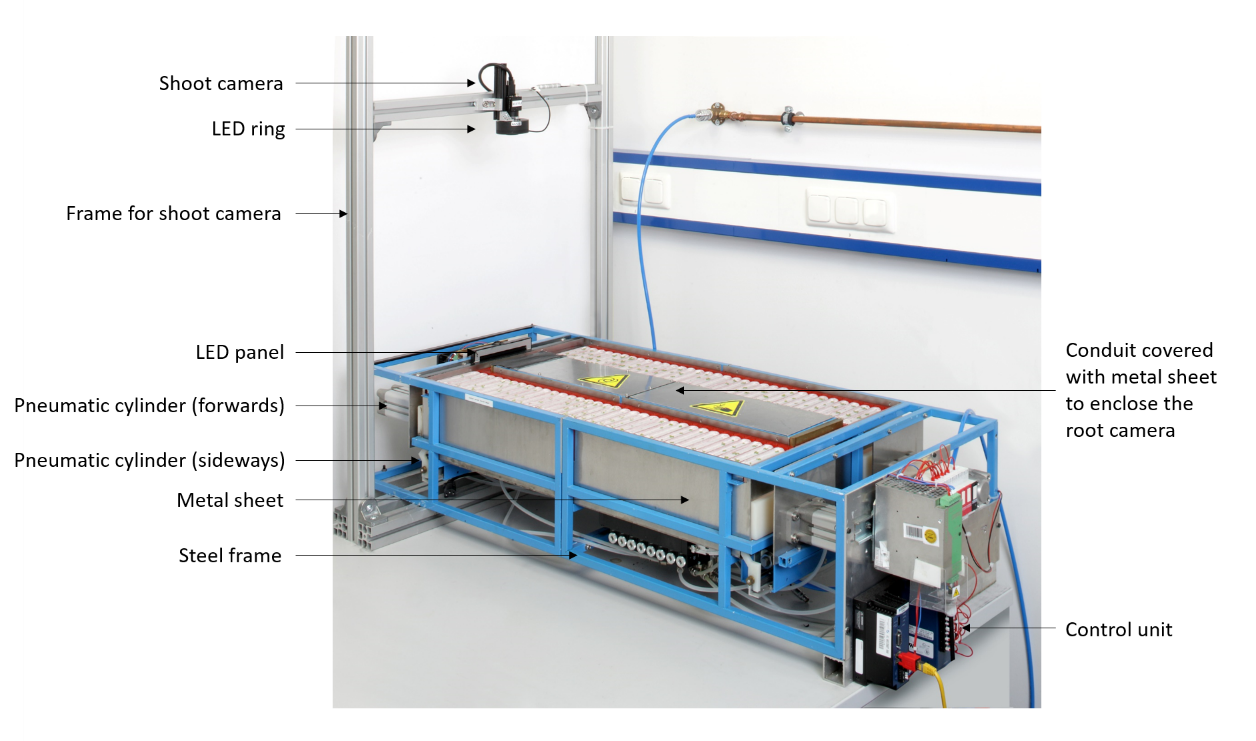


Fig. S3: Parts of *GrowScreen-Agar* setup (see Additional File 1: Table S1).


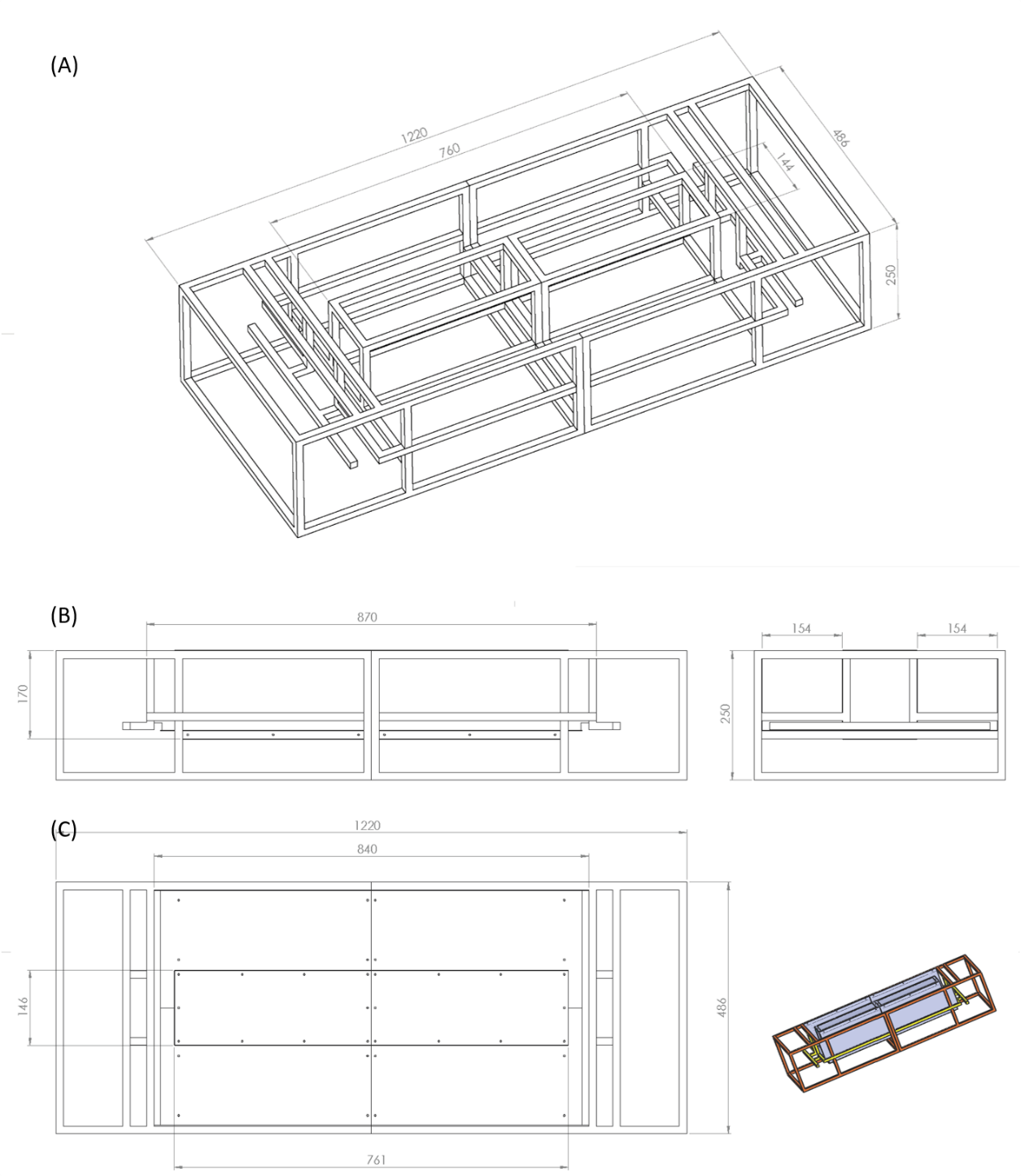


Fig. S4: Steel frame of *GrowScreen-Agar* mechanical setup without (A) and with metal sheets (B – side views and C – top view).


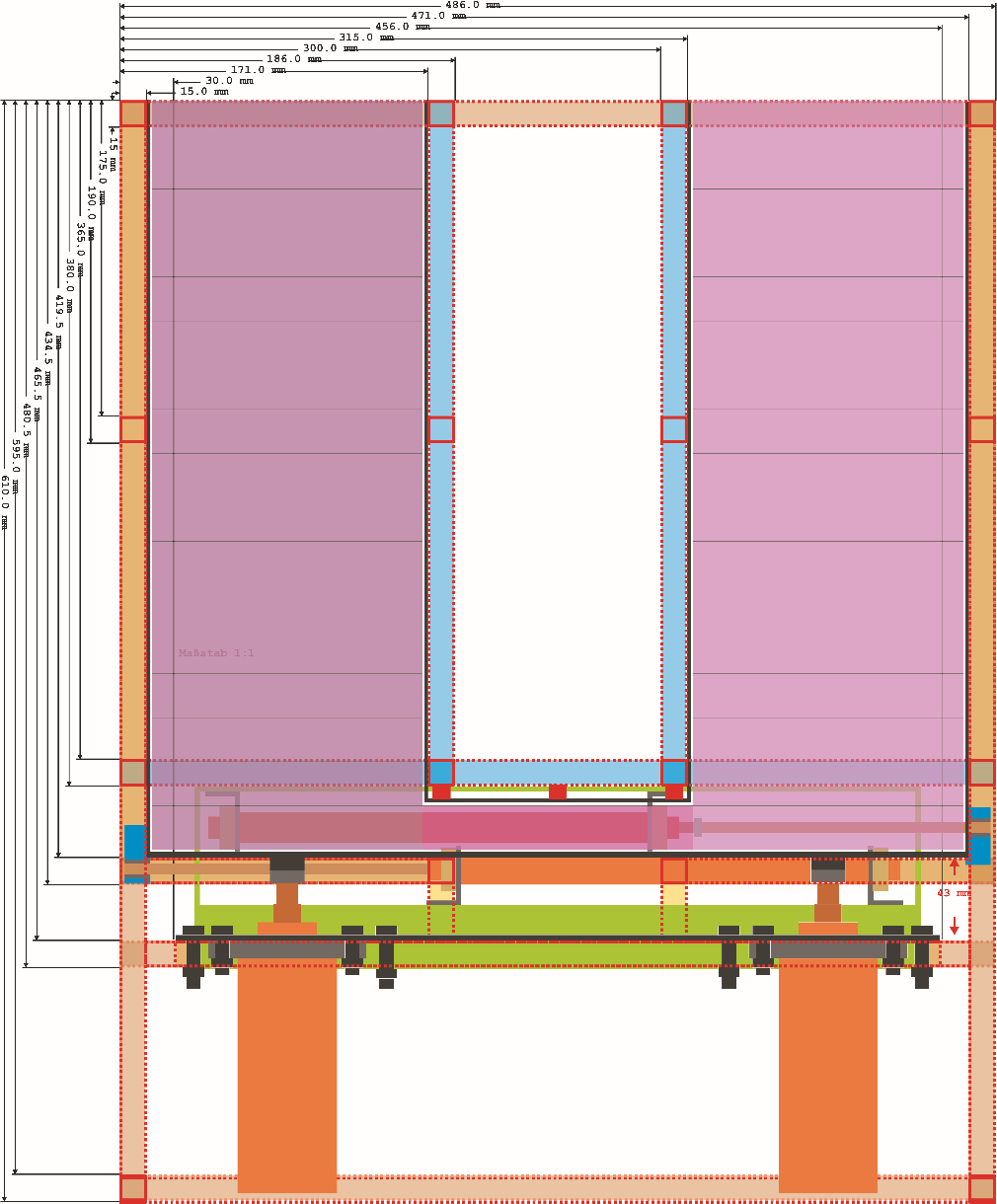

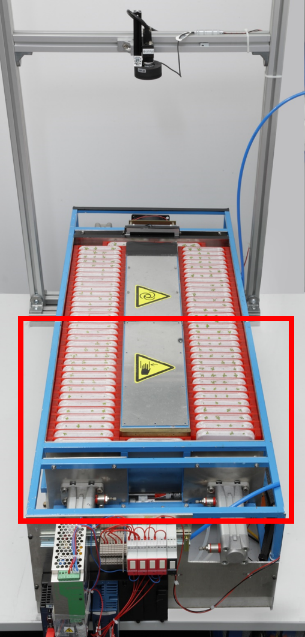


Fig. S5: Top view of *GrowScreen-Agar* mechanical setup. The system consists of two halves which are constructed identical, but only mirrored. The technical drawing (left) shows one half of the system which is marked in red in the original image (right). Pneumatic cylinders (orange parts in front in the technical drawing) are mounted at the edges of the rectangle to push the U-shaped holders forward. The system can push the holders in both directions. The left cylinder is used for pushing the holders clockwise, the right cylinder for counterclockwise. In our study we used only the right cylinder and the counterclockwise rotation of the plates.


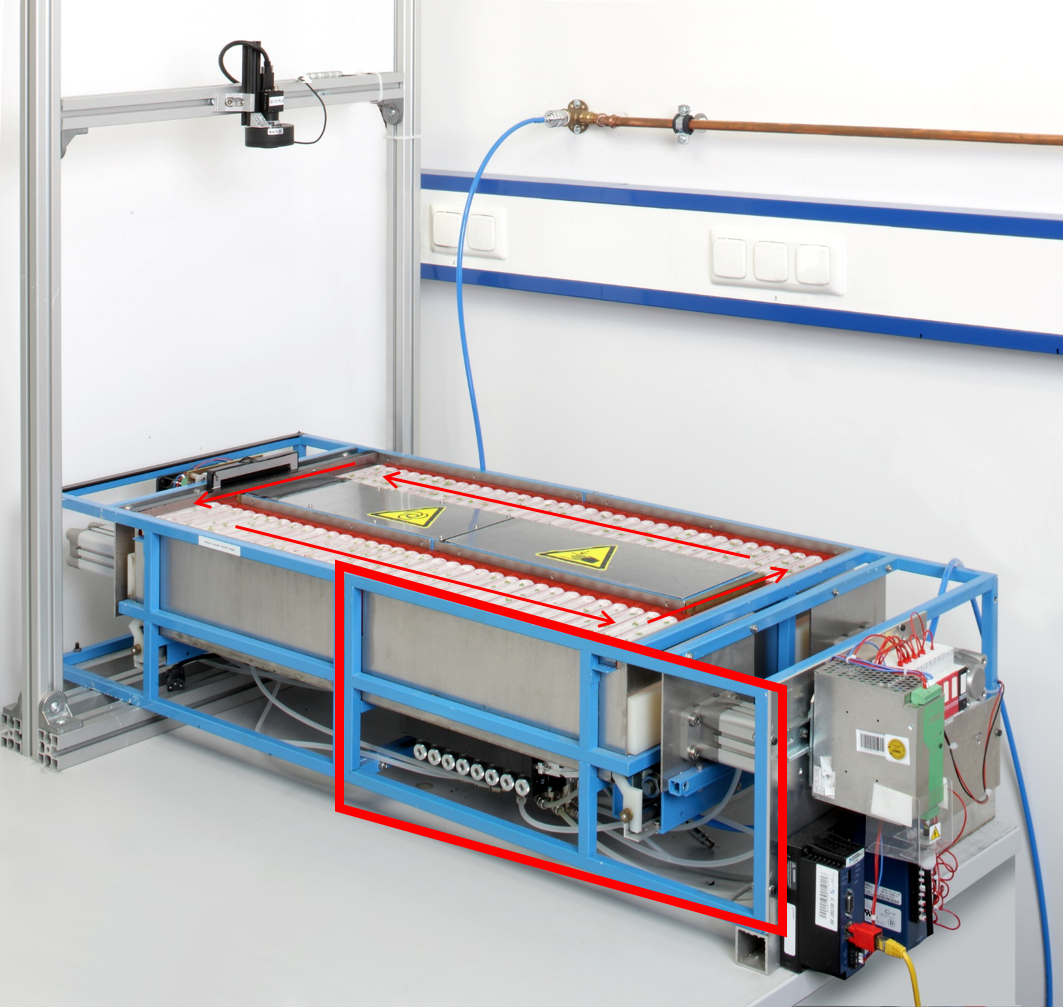


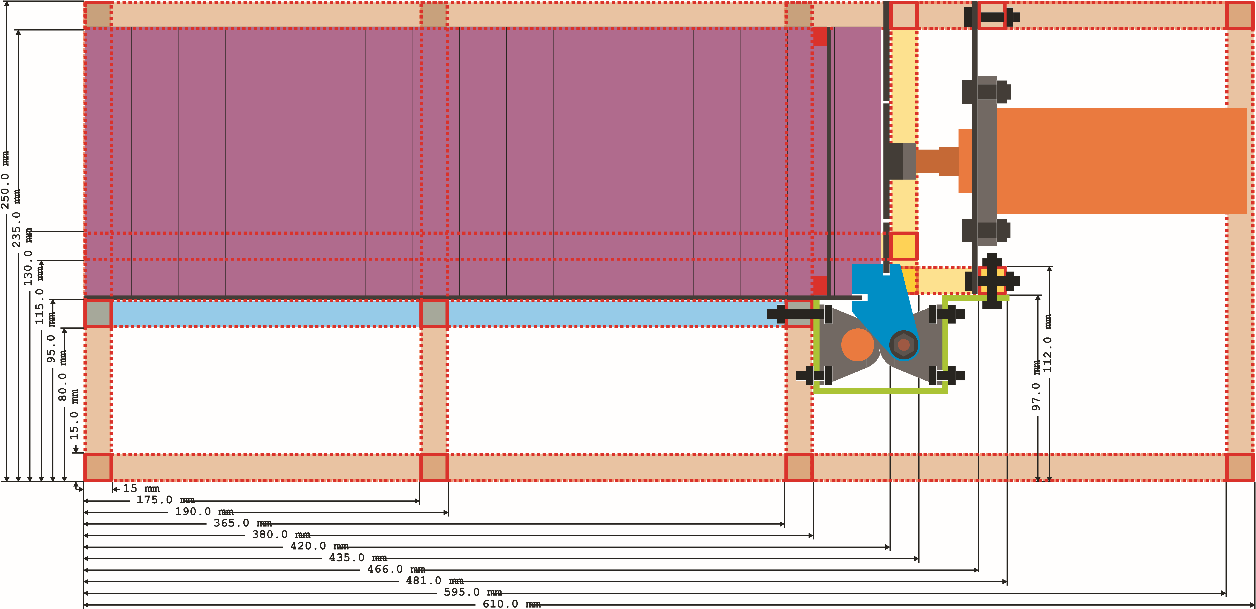


Fig. S6: Side view of *GrowScreen-Agar* mechanical setup. The system consists of two halves which are constructed identical, but only mirrored. The technical drawing (bottom) shows one half of the system which is marked in red in the original image (top). Pneumatic cylinders (orange) are mounted at the edges of the rectangle to push the U-shaped holders forward. The cylinders and its attachments (drawn in blue/grey/orange) which are located below the path of the holders moves two holders sideways at a time. The right cylinder (with visible attachment) is used for counterclockwise rotation of the plates (indicated by the red arrows in the picture at the top). The other cylinders (not used in this study) would push a whole stack of plates clockwise.
